# 3-Deazaguanosine inhibits SARS-CoV-2 viral replication and reduces the risk of COVID-19 pneumonia in hamster

**DOI:** 10.1016/j.isci.2025.112140

**Published:** 2025-03-01

**Authors:** Noriko Saito-Tarashima, Takaaki Koma, Naoto Hinotani, Keigo Yoshida, Moka Ogasa, Akiho Murai, Syuya Inoue, Tomoyuki Kondo, Naoya Doi, Koichi Tsuneyama, Masako Nomaguchi, Noriaki Minakawa

**Affiliations:** 1 Graduate School of Pharmaceutical Science, Tokushima University, 1-78-1 Shomachi, Tokushima, Tokushima 770-8505, Japan; 2 Department of Microbiology, Graduate School of Medicine, Tokushima University, 3-18-15 Kuramoto, Tokushima, Tokushima 770-8503, Japan; 3 Department of PostLED Photonics Research, Institute of PostLED Photonics, Tokushima University, 2-1 Minamijosanjima, Tokushima, Tokushima 770-8506, Japan; 4 Department of Pathology and Laboratory Medicine, Graduate School of Medicine, Tokushima University, 3-18-15 Kuramoto, Tokushima, Tokushima 770-8503, Japan

**Keywords:** Health sciences, Biological sciences, Immunology

## Abstract

The COVID-19 pandemic highlighted the serious threat that coronaviruses have on public health. Because coronavirus continuously undergoes cross-species transmission, additional therapeutic agents and targets are urgently needed. Here, we show that a 3-deazapurine ribonucleoside, 3-Deazaguanosine (C^3^Guo, **2**), has potent antiviral activity against severe acute respiratory syndrome coronavirus 2 (SARS-CoV-2). Unexpectedly, C^3^Guo (**2**) does not act as an inhibitor of RNA-dependent RNA polymerase (RdRp), which is the therapeutic target of two key nucleoside/nucleotide inhibitors approved for the treatment of COVID-19 (Remdesivir and Molnupiravir); instead, it seems to function by targeting the capping machinery of viral RNA. In hamsters infected with SARS-CoV-2, administration of **2** markedly reduced infectious viral titers, and prevented the development of COVID-19 pneumonia better than Molnupiravir. The potency of **2** against SARS-CoV-2 underscores its potential as an effective therapeutic agent for COVID-19 and future zoonotic coronavirus infections and raises the possibility of antiviral nucleoside analogs with alternative therapeutic targets to RdRp.

## Highlights

• 3-Deazaguanosine (C^3^Guo, **2**) suppresses SARS-CoV-2 replication *in vitro* and *in vivo*
• C^3^Guo (**2**) prevents COVID-19 pneumonia better than Molnupiravir in hamster models
• C^3^Guo (**2**) acts independently of RdRp, unlike Remdesivir and Molnupiravir
• C^3^Guo (**2**) is a promising an anti-COVID-19 agent with a unique antiviral mechanism

## Graphical abstract

**Figure:**
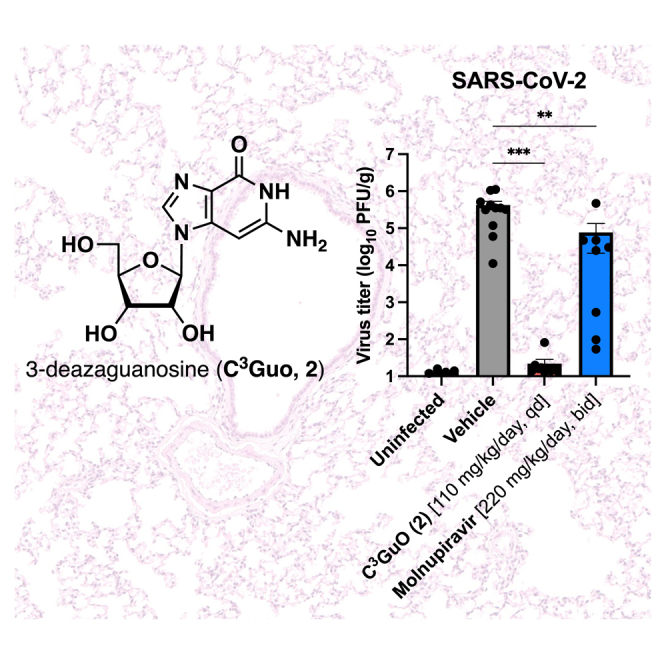

## Introduction

**Figure 1:**
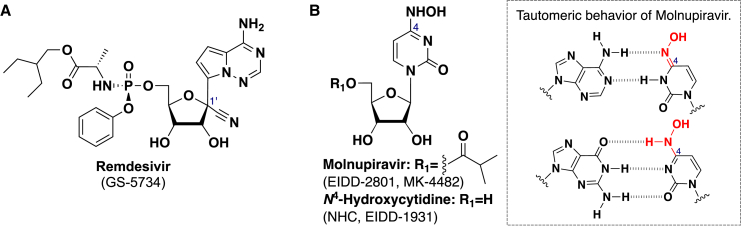
Chemical structures of nucleoside and nucleotide inhibitors (NIs) clinically approved for the treatment of COVID-19 Chemical structure of (A) Remdesivir and (B) Molnupiravir and its parental nucleoside, NHC.

The COVID-19 pandemic, caused by severe acute respiratory syndrome coronavirus 2 (SARS-CoV-2), has dominated world headlines since this positive-strand RNA virus emerged in late 2019. Within 2 years of the first outbreak, an astonishing 10 vaccines were developed and approved, leading to worldwide vaccination and significant success in controlling infection and exacerbation.^1^ In addition to vaccines, numerous drug discovery programs also emerged with some success. The first United States Food and Drug Administration (FDA)-authorized drug to treat COVID-19 was Remdesivir (GS-5734) (Figure 1A), a nucleotide prodrug that targets the RNA-dependent RNA polymerase (RdRp) of SARS-CoV-2.^2^^,^^3^ Remdesivir is a repositioned drug, originally evaluated in clinical trials for treatment of Ebola virus disease, and has broad-spectrum antiviral activity.^4^ Subsequently, Molnupiravir (EIDD-2801; also known as MK-4482) (Figure 1B), a nucleoside prodrug of *N*^4^-hydroxycytidine (NHC, EIDD-1931), was developed and approved as the first orally active COVID-19 drug.^5^ Molnupiravir also targets RdRp of SARS-CoV-2.^6^^,^^7^ More recently, nirmatrelvir (PF-07321332), an inhibitor of the main protease (Mpro) of SARS-CoV-2, was authorized by the FDA in 2021.^8^^,^^9^

Among the various drug targets in RNA viral infections, RdRp is a focus for almost all RNA viruses. Indeed, inhibition of RdRp has become an integrated approach to managing several viral infections, including SARS-CoV-2, hepatitis C, influenza, and dengue viruses, among others.^10^^,^^11^ Nucleoside and nucleotide analog inhibitors (NIs) are an important class of RdRp inhibitors, as evidenced in the early application of Remdesivir and Molnupiravir during the COVID-19 pandemic.^12^ After conversion to the corresponding 5′-triphosphate, NIs are incorporated into the growing viral RNA chain and inhibit viral replication mainly by one of two mechanisms: (1) chain termination and disruption of subsequent viral RNA replication or transcription; or (2) mutagenesis owing to mispairing with and/or substitution of natural nucleotides, leading to impaired RNA synthesis or structure, RNA-protein interactions, or protein functions. Of the two NIs approved as COVID-19 drugs, Remdesivir functions by the chain-termination mechanism; that is, the active 5′-triphosphate of Remdesivir is incorporated into the growing viral RNA product by RdRp, where it acts as a chain-terminator of further RNA elongation.^13^ The C1′-cyano group in the Remdesivir ribose moiety is critical for antiviral potency^14^: steric hindrance between this cyano group and the SARS-CoV-2 RdRp complex acts as a translocation barrier on the growing RNA strand, causing RdRp to stall (Figure 1A).^13^^,^^15^ On the other hand, Molnupiravir mainly acts via mutagenesis. After incorporation in the viral RNA, its 4-hydroxyamino group forms two tautomers: an amino form that pairs with G and an imino form that pairs with A (Figure 1B).^16^ This tautomeric behavior increases the frequency of transition mutations (G-to-A and C-to-U) in the viral genes, leading to lethal mutagenesis.

In terms of the inhibition of DNA polymerase, strand elongation is greatly inhibited when 3-deaza-2′-deoxyadenosine 5′-triphosphate (dC^3^Ado-TP) lacking the minor groove electron pair is incorporated instead of dATP in primer extension reactions with Taq DNA polymerase.^17^ Similarly, the presence of 3-deaza-2′-deoxyguanosine (dC^3^Guo) at the primer terminus decreases the rate of dNTP incorporation by the Klenow fragment DNA polymerase.^18^ These observations suggest that, if metabolized to active 5′-triphosphates and incorporated into the growing DNA strand, dC^3^Ado and dC^3^Guo function as chain terminators for DNA polymerase. However, whether the corresponding 3-deazapurine ribonucleosides/ribonucleotides act as chain terminators for RdRp, has not been elucidated, despite their potential utility as anti-RNA viral agents.^19^^,^^20^

**Figure 2:**
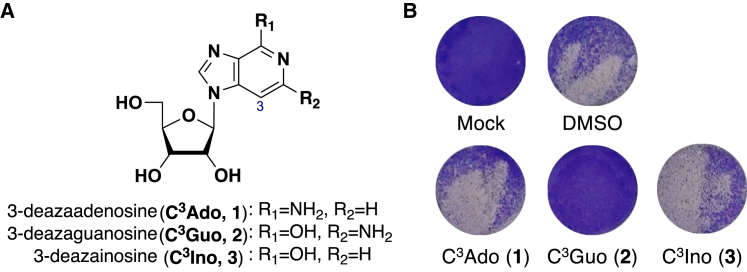
Anti-SARS-CoV-2 activity of 3-Deazapurine ribonucleosides **1**–**3** (A) Structures of 3-deazaadenosine (C^3^Ado, **1**), 3-Deazaguanosine (C^3^Guo, **2**), and 3-deazainosine (C^3^Ino, **3**). (B) CPE assays. Vero E6 cells pre-treated with the indicated compounds (30 μM) were infected with SARS-CoV-2 at a multiplicity of infection (MOI) of 0.001. On day 3 post-infection, cells were fixed and stained with crystal violet to assess CPE. Viable attached cells were stained purple; dead cells due to infection were not stained as they had detached from the wells. Representative data from three independent assays are shown. Mock, an uninfected control; DMSO, an infected control without any compound.

In this study, therefore, we prepared 3-deazapurine ribonucleosides and explored their effects on RdRp of SARS-CoV-2. Although we found that 3-Deazaguanosine (C^3^Guo, **2**) (Figure 2) has potent anti-SARS-CoV-2 activity, contrary to our expectations its triphosphate **2-TP** did not terminate RNA elongation catalyzed by RdRp of SARS-CpV-2; instead, it seemed to function by targeting the capping machinery of viral RNA. In SARS-CoV-2-infected hamsters, administration of **2** significantly decreased SARS-CoV-2 viral titer, and blocked the development of COVID-19 pneumonia better than the existing clinical drug, Molnupiravir. The potency of **2** against SARS-CoV-2 underscores its potential as an effective therapeutic agent for COVID-19 and future zoonotic coronavirus infections and paves the way for antiviral nucleoside analogs with alternative therapeutic targets to RdRp.

## Results and discussion

### C^3^Guo (2) has antiviral activity against SARS-CoV-2

Using our previously reported practical synthesis of 3-deazapurine nucleosides,^21^^,^^22^ we first prepared three kinds of 3-deazapurine ribonucleosides, 3-deazaadenosine (C^3^Ado, **1**), 3-Deazaguanosine (C^3^Guo, **2**), and 3-deazainosine (C^3^Ino, **3**) (Figure 2), and evaluated their inhibitory activity against SARS-CoV-2 by cytopathic effect (CPE) assay (Figures 2 and S1). In this assay, host cell viability, visualized by crystal violet staining, is surrogate readout for viral replication. Of the three 3-deazapurine ribonucleosides prepared, CPE assay clearly showed that guanosine analog **2** inhibited viral replication in Vero E6 cells, strongly suggesting that **2** has effective anti-SARS-CoV-2 activity. In contrast, the other two analogs had limited effect. Real-time quantitative reverse-transcription PCR (RT-qPCR) analysis of viral RNA further confirmed that **2** suppressed SARS-CoV-2 replication in a dose-dependent manner (Figure S2), with its half-maximal effective concentration (EC_50_) was determined to be 1.14 μM. Remdesivir and NHC, the parental analog of Molnupiravir, were included as control compounds, yielding EC_50_ values of 0.47 μM and 0.27 μM, respectively, both consistent with reported literature values (Table 1).^6^^,^^23^ Additionally, the half-maximal cytotoxic concentration (CC_50_) of **2** was sufficiently high at >200 μM for Vero E6 cells (Table 1).

**Table 1:** Anti-SARS-CoV-2 activity and cytotoxicity of 3-deazapurine derivatives **1**–**6**, **10,** and **11**


Compound	R_1_	R_2_	R_3_	R_4_	EC_50_ (μM) (95% CI)	CC_50_ (μM)^c^
3-deazaadenosine (C^3^Ado, **1**)	–NH_2_	–H	–H	–OH	>30^a^	>200
3-Deazaguanosine (C^3^Guo, **2**)	–OH	–NH_2_	–H	–OH	1.14^b^ (0.44–3.34)	>200
3-deazainosine (C^3^Ino, **3**)	–OH	–H	–H	–OH	>30^a^	>200
3-fluoro-3-Deazaguanosine (**4**)	–OH	–NH_2_	–F	–OH	>30^a^	>200
3-chloro-3-Deazaguanosine (**5**)	–OH	–NH_2_	–Cl	–OH	>30^a^	>200
3-deazadiaminopurine ribonucleoside (**6**)	–NH_2_	–NH_2_	–H	–OH	>30^a^	>200
3-deazaguanine (C^3^Gua, **10**)	–	–	–	–	12.6^b^ (3.51–161)	>200
2′-deoxy-3-Deazaguanosine (dC^3^Guo, **11**)	–OH	–NH_2_	–H	–H	5.35^b^ (1.62–32.7)	>200
Remdesivir (GS-5734)	–	–	–	–	0.47^b^ (0.30–0.75)	>200
*N*^4^-hydroxy cytidine (NHC, EIDD-1931)	–	–	–	–	0.27^b^ (0.14–0.59)	>200

a Anti-SARS-CoV-2 activity was evaluated by CPE assay using SARS-CoV-2-infected Vero E6 cells.

b Anti-SARS-CoV-2 activity was evaluated by qRT-PCR of viral RNA using SARS-CoV-2-infected Vero E6 cells. Each EC_50_ value was calculated by non-linear curve fit analysis using GraphPad Prism software.

c Cytotoxicity was evaluated by WST-8 assay using uninfected Vero E6 cells.

**Scheme 1:**
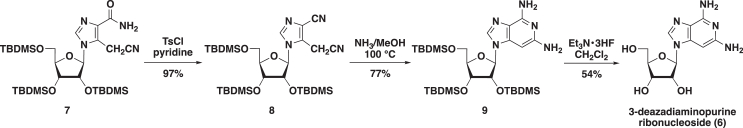
Chemical synthesis of 3-deazadiaminopurine ribonucleoside (**6**) TBDMS, *tert*-butyldimethylsilyl; TsCl, *p*-toluenesulfonyl chloride.

To probe the structure-activity relationship, we evaluated the anti-SARS-CoV-2 activity of two C^3^Guo analogs with halogen atoms at the *C*3 position, **4**^24^ and **5**,^24^ that we previously developed. Substitution of the *C*3 hydrogen of **2** with fluoride (**4**) or chloride (**5**) led to a loss of anti-viral activity, suggesting the *C*3 hydrogen atom is critical for exerting an inhibitory effect (Table 1). Next, we prepared 3-deazadiaminopurine ribonucleoside (**6**) (Scheme 1), which might be converted to **2** by 6-deamination by endogenous adenosine deaminase, and evaluated its anti-SARS-CoV-2 activity (Table 1). Starting with **7**,^21^ a common synthetic intermediate for **2**, dehydration of 4-carboxamide group with *p*-toluenesulfonyl chloride (TsCl) afforded the di-cyano derivative **8**. Subsequent treatment with methanolic ammonia under heating facilitated cyclization to give the protected 3-deazadiaminopurine derivative **9** in 77% yield. The desired 3-deazadiaminopurine ribonucleoside (**6**) was ultimately obtained when **9** was treated with triethylamine trihydrofluoride (Et_3_N･3HF); however, the resulting compound **6** showed no inhibitory effects on SARS-CoV-2 (Table 1). It has been previously reported C^3^Ado (**1**) has resistance to adenosine deaminase.^25^ In the case of **6**, therefore, the substitution of *N*3 for *C*3 might similarly confer resistance to 6-deamination.

For their antiviral effect, NIs generally rely on cellular kinases to undergo stepwise addition of phosphate groups to form the corresponding active nucleoside 5′-triphosphate.^26^ Similarly, C^3^Guo (**2**) may also depend on cellular kinases for stepwise phosphorylation to give its active form, C^3^Guo (**2**) 5′-triphosphate (**2-TP**) (Figure S3A).^27^ Additionally, it is possible that **2** would be first phosphorolysed to 3-deazaguanine (C^3^Gua, **10**) and d-ribose 1-phosphate by purine nucleoside phosphorylase (Figure S3B). The resulting C^3^Gua (**10**) would be then converted into C^3^Guo (**2**) 5′-monophosphate (**2-MP**) via glycosylation with phosphoribosyl pyrophosphate catalyzed by hypoxanthine guanine phosphoribosyl transferase.^28^ Finally, subsequent stepwise phosphorylation of **2-MP** would yield an active 5′-triphosphate, **2-TP**.

**Scheme 2:**
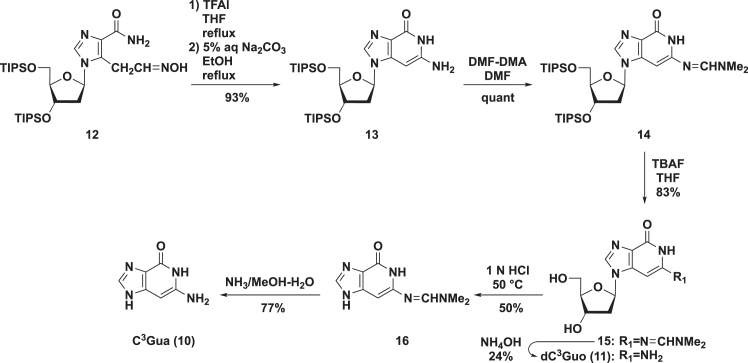
Chemical synthesis of C^3^Gua (**10**) and dC^3^Guo (**11**) TIPS, triisopropylsilyl; TFAI, trifluoroacethyl imidazole; THF, tetrahydrofuran; DMF-DMA, *N*,*N*-dimethylformamide dimethyl acetal; TBAF, tetrabutylammonium fluoride.

Based on this prediction, we evaluated the anti-SARS-CoV-2 activity of C^3^Gua (**10**)^29^ and 2′-deoxy-3-Deazaguanosine (dC^3^Guo, **11**) (Scheme 2),^30^ which were chemically synthesized as follows. Starting with **12**,^31^ dehydration with trifluoroimidazole (TFAI) followed by cyclization with aqueous Na_2_CO_3_ in EtOH under heating afforded the dC^3^Guo derivative **13**. Initially, we tried deprotection of the triisopropylsilane (TIPS) groups on the sugar moiety of **13**, but purification of the resulting compounds was difficult; therefore, we protected the exocyclic amino group of **13** with a dimethylaminomethylene group to give **14**. The TIPS groups were then deprotected with tetrabutylammonium fluoride (TBAF), and the resulting **15** was gently heated in 1 N HCl to give **16**. Treatment of **16** with methanolic ammonia ultimately afforded the desired C^3^Gua (**10**) in good yield. The same steps were followed to prepare dC^3^Guo (**11**), except that **15** was treated with ammonium hydroxide. By CPE assay in Vero E6 cells, the resulting C^3^Gua (**10**) and dC^3^Guo (**11**) both had anti-SARS-CoV-2 activity, as expected (data not shown). Furthermore, the EC_50_ values of **10** and **11**, based on measured viral titers in SARS-CoV-2 infected Vero E6 cells, were 12.6 μM and 5.35 μM, respectively. These findings suggested that **2-TP** serves as the active species responsible for the antiviral activity of C^3^Guo (**2**).

### C^3^Guo-TP (2-TP), the predicted active metabolite of C^3^Guo (2), does not act as a chain terminator of RdRp, but significantly reduces 5′-capping of viral RNA

**Scheme 3:**
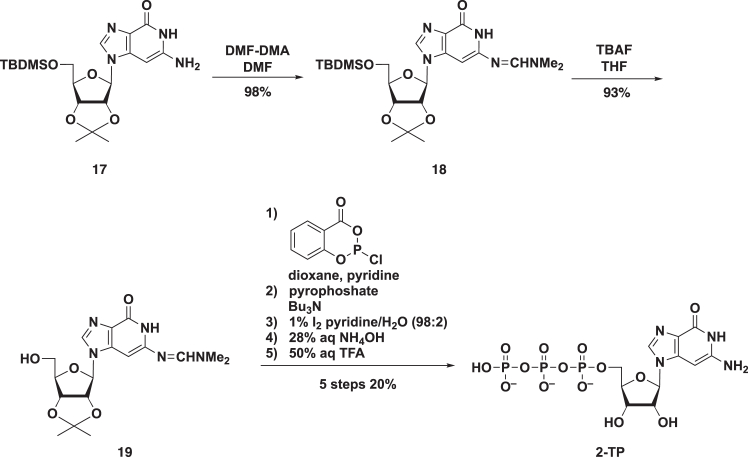
Chemical synthesis of C^3^Guo (**2**) 5′-triphosphate (**2-TP**) TBDMS, *tert*-butyldimethylsilyl; TFA, trifluoroacetic acid; TBAF, tetrabuthylammonium fluoride.

Next, to verify that C^3^Guo (**2**) exerts anti-SARS-CoV-2 activity as a chain terminator of RdRp, we synthesized C^3^Guo-TP (**2-TP**), which, as discussed above, is predicted to be the active metabolite of **2** (Scheme 3). Starting with **17**,^22^ the exocyclic amino group was protected with a dimethylaminomethylene group to give **18**. After the deprotection of *tert*-butyldimethylsilyl (TBDMS) group with TBAF, the resulting **19** was used as a substrate for triphosphate synthesis. Based on a previous method for producing 5′-triphosphate,^32^ **19** was treated with 2-chloro-4*H*-1,3,2-benzodioxaphosphorin-4-one, followed by bis(tri-*n*-butylammonium)pyrophosphate to give the cyclotriphosphite intermediate. This intermediate was then oxidized by 1% iodine in pyridine–H_2_O, followed by successive treatment with NH_4_OH and then 50% aq. trifluoroacetic acid (TFA) to give the desired product **2-TP** in five steps and in 20% yield after purification on a DEAE Sephadex column.

**Figure 3:**
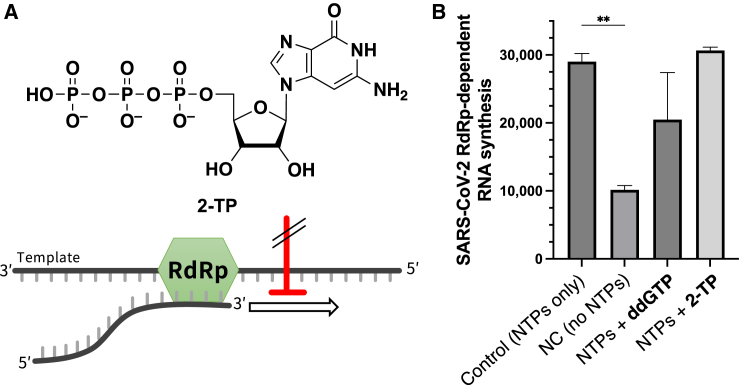
An active metabolite of C^3^Guo (**2**), **2-TP**, is not a chain terminator of SARS-CoV-2 RdRp (A) Schematic of the SARS-CoV-2 RdRp-dependent RNA synthesis assay. (B) Results of the SARS-CoV-2 RdRp-dependent RNA synthesis. The reactions comprised RNA template–product scaffold, recombinant SARS-CoV-2 RdRp complex, NTPs, and 10 μM ddGTP or **2-TP**. After incubation at 34°C for 2 h, fluorescent intercalating dye was added. SARS-CoV-2 RdRp-dependent RNA synthesis levels were determined from fluorescence intensity (Ex, 485 ± 5 nm; Em, 535 ± 10 nm) mean. All experiments were performed in quadruplicate; data are presented as mean + SEM. Statistically significant differences against the control reaction were determined using one-way ANOVA followed by Dunnett’s multiple comparisons test: ns (not significant), ∗∗*p* ≤ 0.01. NC, negative control.

We tested **2-TP** in an *in vitro* SARS-CoV-2 RdRp-dependent RNA synthesis assay (Figure 3A). In this assay, the reactions comprised an RNA template-product scaffold, recombinant SARS-CoV-2 RdRp complex, NTPs, and **2-TP**. A fluorescent intercalating dye was then added after incubation to determine the levels of RNA synthesis based on fluorescence intensity. Unexpectedly, RNA synthesis by the recombinant SARS-CoV-2 RdRp complex was not inhibited by the addition of **2-TP**, whereas it was reduced by 2′,3′-dideoxy GTP (ddGTP), a potential chain terminator of viral RNA synthesis^33^ (Figure 3B). This observation suggested that **2-TP** does not act as a chain terminator of SARS-CoV-2 viral RNA replication, contradicting our initial prediction that 3-deazapurine ribonucleosides might inhibit SARS-CoV-2 RdRp.

To further elucidate the mechanism underlying the anti-SARS-CoV-2 activity of C^3^Guo (**2**), we next considered the capping pathway as a potential target of the active metabolite **2-TP**. To this end, we tested the effect of **2-TP** on the capping reaction catalyzed by vaccinia capping enzyme (VCE), which has been well characterized (Figure S4A).^34^^,^^35^^,^^36^ In the control reaction with model RNA fragment and natural GTP, 5′-capping and subsequent *N*7 methylation by VCE proceeded efficiently to give 5′-m7GpppG capped RNA (**IVa**), as determined by liquid chromatograph-mass spectrometry (LC-MS) (Figure S4B), while no obvious capped RNA (**IIIa** or **IVa**) was observed in the reaction without GTP (Figure S4C). When **2-TP** was included in the reaction, it was incorporated into the 5′-cap structure, yielding small amounts of 5′-C^3^GpppA-capped RNA (**IIIb**) and 5′-m7C^3^GpppG-capped RNA (**IVb**) (Figure S4D). Furthermore, in the presence of both GTP and **2-TP**, the capping reactions yielded no 5′-capped RNA (**IIIa,b** or **IVa,b**) and the RNA substrates (**I**) were degraded (Figure S4E). Although this is only one possibility because viruses have different capping machinery and various molecular recognition mechanisms,^37^ these data suggest that C^3^Guo (**2**) exerts its antiviral activity by targeting the capping machinery of SARS-CoV-2 rather than by targeting RdRp.

### C^3^Guo (2) reduces viral titer and inhibits COVID-19 pneumonia in hamsters infected with SARS-CoV-2

**Figure 4:**
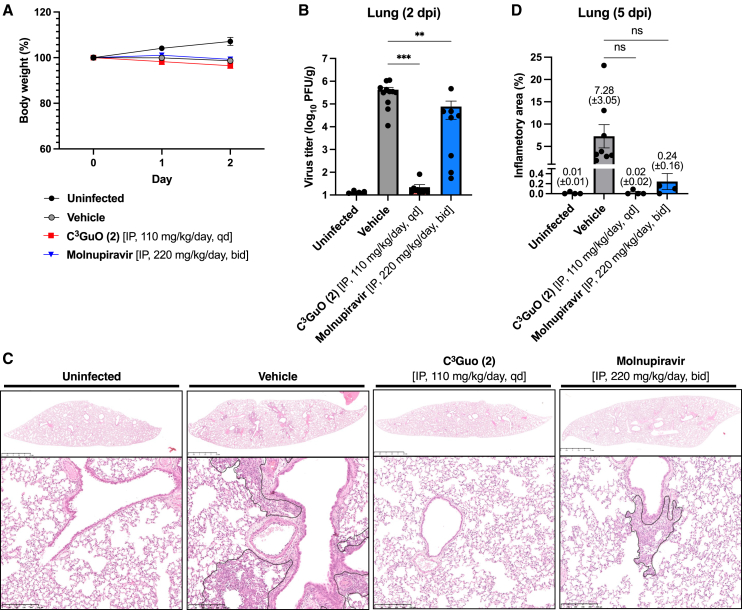
Intraperitoneal treatment of C^3^Guo (**2**) controls viral burden and disease in hamsters inoculated with SARS-CoV-2 (A) Body weight changes in uninfected hamsters and SARS-CoV-2-infected hamsters treated with vehicle, C^3^Guo (**2**), or Molnupiravir are shown. (B) Viral titer of lung samples at 2 dpi. (C and D) Lung histopathology at 5 dpi. (C) Representative data from four or more independent assays are shown. The area of inflammation was determined (black line, lower panels), and (D) the percentage of inflammation in the lung sections was calculated. Groups of uninfected (*n* = 4) and SARS-CoV-2-infected hamsters treated with vehicle (*n* = 8), C^3^Guo (**2**) (*n* = 4), or Molnupiravir (*n* = 4) were shown. Horizontal bars represent the mean value of each group. Statistically significant differences against the vehicle control were determined using one-way ANOVA, followed by Dunnett’s multiple comparisons tests: ns (not significant), ∗∗*p* ≤ 0.01, and ∗∗∗*p* ≤ 0.001. IP, intraperitoneal.

To further assess the effect of C^3^Guo (**2**) on SARS-CoV-2 infection *in vivo*, we initially conducted a repeated dose toxicity study in C57BL/6J mice. Intraperitoneal (IP) administration of C^3^Guo (**2**) to mice at a concentration of 12.5 mg/kg/day or 112.5 mg/kg/day for 5 consecutive days led to no abnormal body weight changes or clinical observations, indicating that **2** does not cause adverse effects in mammals (Figure S5). Next, we investigated the *in vivo* antiviral activity of **2** using a Syrian hamster model of COVID-19.^38^^,^^39^ Hamsters were inoculated with SARS-CoV-2 (1 × 10^2^ PFU) and, starting 2 h before infection, given daily IP doses of **2** at 110 mg/kg/day (qd). Control hamsters (0 mg/kg/day) received a daily dose of saline IP as the vehicle alone. Molnupiravir (220 mg/kg/day) was used as the primary comparator drug and given twice daily (bid), following a previously established protocol,^40^^,^^41^ as it demonstrated superior anti-SARS-CoV-2 compared to Remdesivir in our *in vitro* evaluation, as shown in Table 1. Whereas uninfected animals gained weight, animals in the infected groups showed a marginal reduction in body weight regardless of treatment with C^3^Guo (**2**), Molnupiravir, or the vehicle (Figure 4A). At 2 days post-infection (dpi), when viral load reaches its peak, the titer in lungs of hamsters treated with antiviral drugs (**2** or Molnupiravir) was lower than that in those of vehicle controls (Figure 4B). Notably, treatment with **2** (110 mg/kg/day) resulted in more than a 10^5^-fold lower infectious viral titer, comparable to the levels observed in uninfected controls.

Histopathological analysis of lung tissues using hematoxylin and eosin staining at 5 dpi showed that severe pneumonia was evident in the vehicle control group, with inflammation observed in 7.28 ± 3.05% of lung sections (Figures 4C and 4D). Conversely, inflammation in lungs from animals treated with C^3^Guo (**2**) was notably low (0.02 ± 0.02%) and comparable to that observed in the uninfected group (0.01 ± 0.01%); even though lung histopathology from the Molnupiravir-treated group showed mild pneumonia (0.24 ± 0.16%). Collectively, these findings demonstrate that IP treatment with C^3^Guo (**2**) significantly inhibits viral replication and prevents the development of pneumonia in SARS-CoV-2-infected hamsters.

**Figure 5:**
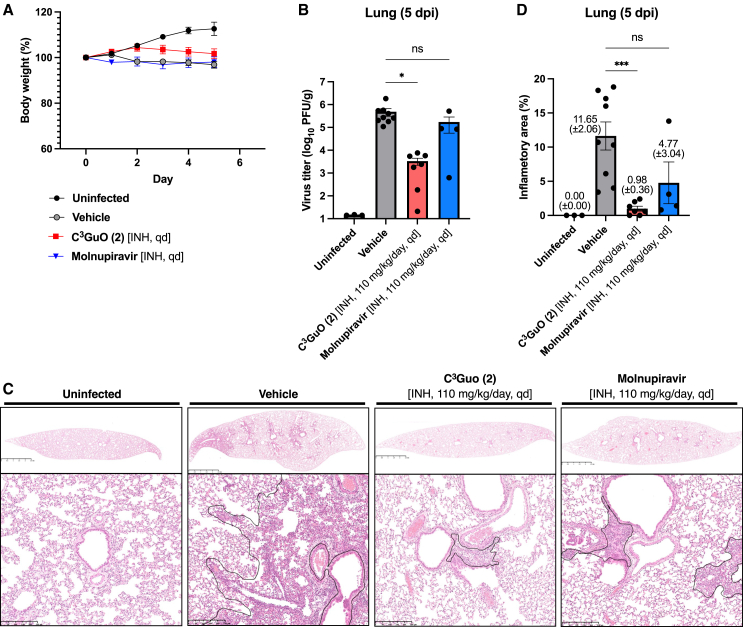
Inhalation treatment of C^3^Guo (**2**) controls viral burden and disease in hamsters inoculated with SARS-CoV-2 (A) Body weight changes in uninfected hamsters and SARS-CoV-2-infected hamsters treated with vehicle, C^3^Guo (**2**), or Molnupiravir are shown. (B) Viral titer of lung samples at 5 dpi. (C and D) Lung histopathology at 5 dpi. (C) Representative data from four or more independent assays are shown. The area of inflammation was determined (black line, lower panels), and (D) the percentage of inflammation in the lung sections was calculated. Groups of uninfected (*n* = 3) and SARS-CoV-2-infected hamsters treated with vehicle (*n* = 9), C^3^Guo (**2**) (*n* = 7), or Molnupiravir (*n* = 4) were shown. Horizontal bars represent the mean value of each group. Statistically significant differences against the vehicle control were determined using one-way ANOVA, followed by Dunnett’s multiple comparisons tests: ns (not significant), ∗*p* ≤ 0.05, and ∗∗∗*p* ≤ 0.001. INH, inhalation.

We also tested the *in vivo* anti-SARS-CoV-2 activity of C^3^Guo (**2**) when administered by inhalation using a nebulizer, with a view to exploring its potential clinical application (Figure 5). To simplify the comparison of antiviral and pneumonia-preventive effects, the same doses of Molnupiravir and C^3^Guo (**2**) were used. Thus, nebulizers were filled with saline or 10 mg/mL of antiviral drug (**2** or Molnupiravir) to a volume corresponding to 170 mg/kg/day; they were inhaled by the hamsters once a day (qd) until no aerosol was produced. Based on the residual drug in the nebulizer, the estimated aerosolized inhalation dose was approximately 110 mg/kg/day for either drug.

Similar levels of body weight loss associated with infection were observed in the vehicle-treated control group and the Molnupiravir-treated group (Figure 5A). In contrast, hamsters treated with C^3^Guo (**2**) showed a reduction in the body weight loss associated with infection. Furthermore, inhalation of **2** resulted in more than 10^2^-fold lower infectious viral titer in the lungs of hamsters at 5 dpi (Figure 5B). Whereas histopathological findings of viral pneumonia were pronounced in the vehicle group (11.65 ± 2.06%), C^3^Guo (**2**) significantly attenuated the lung pathology, reducing the proportion of inflammatory area per lung section to 0.98 ± 0.36% (Figure 5C). These results indicate that inhalation of C^3^Guo (**2**) largely prevented viral pneumonia, making it a promising option for COVID-19 treatment.

### Conclusion

The present study showed that C^3^Guo (**2**) exerts antiviral activity against SARS-CoV-2. Our structure–activity relationship analysis suggested that **2** is intracellularly converted into an active metabolite, **2-TP**. *In vitro* assays of SARS-CoV-2 RdRp-dependent RNA synthesis and the VCE capping reaction showed that **2-TP** does not act as a chain terminator of RdRp, but it significantly reduces 5′-capping of viral RNA. In animals infected with SARS-CoV-2, administration of C^3^Guo (**2**) markedly reduced infectious viral titer to a greater extent than the existing clinical drug Molnupiravir, which was administered more frequently. Furthermore, the development of pneumonia caused by SARS-CoV-2 infection was inhibited by the administration of C^3^Guo (**2**). There have been several reports on the antiviral activity of 3-deazapurine nucleosides, but most of them concern C^3^Ado (**1**), which acts as an inhibitor of *S*-adenosylhomocysteine hydrolase.^19^^,^^42^^,^^43^ The potency of C^3^Guo (**2**) against SARS-CoV-2 underscores its potential as an effective therapeutic agent for COVID-19 and other future zoonotic coronavirus infections and highlights the potential of nucleoside analogs as antiviral agents with alternative therapeutic targets to RdRp.

### Limitations of the study

We showed that C^3^Guo (**2**) has potent antiviral activity against SARS-CoV-2, but its activity against other RNA viruses was not evaluated. Future studies should explore the breadth of activity of C^3^Guo (**2**). We further showed that the active metabolite of C^3^Guo (**2**), **2-TP**, does not act as a chain terminator of SARS-CoV-2 RdRp, but instead seems to target the capping machinery of SARS-CoV-2. However, we did not provide direct evidence of 5′-capping inhibition by **2**. Future experiments should investigate in detail the antiviral mechanism of C^3^Guo (**2**).

## Resource availability

### Lead contact

Further information and requests for resources should be directed to and will be fulfilled by Noriaki Minakawa (minakawa@tokushima-u.ac.jp).

### Materials availability

Compounds in this study will be provided following the request to the lead contact.

### Data and code availability

• Data: All data reported in this paper will be shared by the lead contact upon reasonable request.
• Code: This study did not generate original code.
• Others: Any additional information regarding the data reported in this paper is available from the lead contact upon reasonable request.

## Acknowledgments

We thank Ms. Ayako Matsuo for kindly providing technical assistance for the *in vitr*o experiments. This work was financially supported, in part, by 10.13039/501100001691JSPS KAKENHI grant numbers 21H02606 (N.M.), 24K02149 (N.M.) and 22K06527 (N.S.-T.), the 10.13039/100008732Uehara Memorial Foundation (N.M.), 10.13039/100007449Takeda Science Foundation (T.K.), the 10.13039/501100003837Ichiro Kanehara Foundation (T.K.), 10.13039/100016289Taiju Life Social Welfare Foundation (T.K.), YOKOYAMA Foundation for Clinical Pharmacology grant numbers YRY-2324 (T.K.), and the 10.13039/100007428Naito Foundation (N.S.-T.). N.H., K.Y., M.O., A.M., and S.I. are grateful to the research program for development of the intelligent Tokushima artificial exosome (iTEX) at 10.13039/501100005623Tokushima University.

## Author contributions

N.-S.T. and T. Koma: Contributed equally to study. conceptualization, N.-S.T. and N.M.; methodology, N.-S.T., T. Koma, M.N.; formal analysis, N.S.-T., T. Koma, T.Kondo, and K.T.; investigation, N.S.-T., T. Koma, N.H., K.Y., M.O., A.M., S.I., T. Kondo, N.D., and M.N.; data curation, N.S.-T.; writing – original draft, N.S.-T. and T. Koma; writing – review and editing, N.S.-T., T. Koma, M.N., and N.M.; visualization, N.S.-T. and T. Koma; supervision, N.S.-T., M.N., and N.M. All authors have given approval to the final version of the manuscript.

## Declaration of interests

The authors declare no competing interests.

## STAR★Methods

### Key resources table

**Table:** 

REAGENT or RESOURCE	SOURCE	IDENTIFIER
**Bacterial and virus strains**		

SARS-CoV-2/Hu/DP/Kng/19-020	Kanagawa Prefectural Institute of Public Health	GenBank LC528232

**Chemicals, peptides, and recombinant proteins**		

3-deazaadenosine (C^3^Ado, **1**)	Hinotani et al.^22^	Prepared at the Faculty of Pharmaceutical Science, Tokushima University, and its chemical purity (>95%) was verified by UPLC-MS analysis (see the supplemental information)
3-Deazaguanosine (C^3^Guo, **2**)	Hinotani et al.^22^	Prepared at the Faculty of Pharmaceutical Science, Tokushima University, and its chemical purity (>95%) was verified by UPLC-MS analysis (see the supplemental information)
3-deazainosine (C^3^Ino, **3**)	Hinotani et al.^22^	Prepared at the Faculty of Pharmaceutical Science, Tokushima University, and its chemical purity (>95%) was verified by UPLC-MS analysis (see the supplemental information)
3-fluoro-3-Deazaguanosine (**4**)	Minakawa et al.^24^	Prepared at the Faculty of Pharmaceutical Science, Tokushima University, and its chemical purity (>95%) was verified by UPLC-MS analysis (see the supplemental information)
3-chloro-3-Deazaguanosine (**5**)	Minakawa et al.^24^	Prepared at the Faculty of Pharmaceutical Science, Tokushima University, and its chemical purity (>95%) was verified by UPLC-MS analysis (see the supplemental information)
3-deazadiaminopurine ribonucleoside (**6**)	This study	Prepared at the Faculty of Pharmaceutical Science, Tokushima University, and its chemical purity (>95%) was verified by UPLC-MS analysis (see the supplemental information)
3-deazaguanine (C^3^Gua, **10**)	This study	Prepared at the Faculty of Pharmaceutical Science, Tokushima University, and its chemical purity (>95%) was verified by UPLC-MS analysis (see the supplemental information)
2′-deoxy-3-Deazaguanosine (C^3^Guo, **2**)	This study	Prepared at the Faculty of Pharmaceutical Science, Tokushima University, and its chemical purity (>95%) was verified by UPLC-MS analysis (see the supplemental information)
*N*^4^-hydroxycytidine (NHC, EIDD-1931)	Paymode et al.^44^	Prepared at the Faculty of Pharmaceutical Science, Tokushima University, and its chemical purity (>95%) was verified by UPLC-MS analysis (see the supplemental information)
Remdesivir (GS-5734)	Cayman Chemical Company	Cat#30354
Molnupiravir (EIDD-2801)	Merck & Co., Inc.	LAGEVRIO CapsulesⓇ
C^3^Guo-TP (**2-TP**)	This study	Prepared at the Faculty of Pharmaceutical Science, Tokushima University, and its chemical purity (>95%) was verified by UPLC-MS analysis (see the supplemental information)
ddGTP	BIOLOG Life Science Institute GmbH & Co. KG	Cat#D019-05
DMSO	FUJIFUILM Wako Pure Chemical Corporation	Cat#048-32811
Otsuka Normal Saline	Otsuka Pharmaceutical Co., Ltd.	https://www.otsukakj.jp/med_nutrition/dikj/menu1/000224.php
10% Formalin solution	FUJIFUILM Wako Pure Chemical Corporation	Cat#060-03845
Crystal violet	FUJIFUILM Wako Pure Chemical Corporation	Cat#031-04852
QIAamp Viral RNA Mini kit	Qiagen	Cat#52926
Fast SYBR Green RT-PCR kit	Qiagen	Cat#204156
MEGAshortscript	Thermo Fisher Scientific	Cat#AM1354
10% Phosphate-buffered formalin solution	FUJIFUILM Wako Pure Chemical Corporation	Cat#062-01661
Recombinant SARS-CoV-2 RdRp complex	ProFoldin	Cat#RDRP-100S2

**Critical commercial assays**		

RNA polymerase assay kit	ProFoldin	Cat#S2RPA100K
ScriptCap^TM^ m^7^G Capping System	CellScript	Cat# C-SCCS2250

**Experimental models: Cell lines**		

Vero E6 cells	ATCC	Cat#CRL-1586

**Experimental models: Organisms/strains**		

C57BL/6J (female, 6 weeks old)	CLEA Japan, Inc.	C57BL/6JJcl
Syrian hamster (male, 5 weeks old)	Japan SLC	Slc:Syrian

**Oligonucleotides**		

5′-d(AAATTTTGGGGGACCAGGAAC)-3′	SARS-CoV-2 nucleocapsid	Shintaro et al.^45^ Chemically prepared.
5′-d(TGGCACCTGTGTAGGGTCAAC)-3′	SARS-CoV-2 nucleocapsid	Shintaro et al.^45^ Chemically prepared.
5′-r(gggcgaauuaa)-3′	Ohno et al.^46^	Prepared by *in vitro* transcription.
5′-d(CAGTGAATTGTAATACGACT CACTATAGGGC)-3′	Ohno et al.^46^	Chemically prepared.
5′-d(TTAATTCGCCCTATAGTGAG TCGTATTACAATTCACTG)-3′	Ohno et al.^46^	Chemically prepared.

**Software and algorithms**		

ChemDraw Professional 21.0	PerkinElmer	https://www.perkinelmer.com/category/chemdraw
Delta 6.1.0	JEOL	https://nmrsupport.jeol.com/
GraphPad Prism 9	GraphPad	https://www.graphpad.com/
UNIFI	Waters	https://www.waters.com/waters/en_US/UNIFI-Scientific-Information-System/nav.htm?cid=134801648&lset=1&locale=en_US&changedCountry=Y
7500 Real-Time PCR Software v2.3	Applied Biosystems	https://www.thermofisher.com/jp/ja/home/technical-resources/software-downloads/applied-biosystems-7500-real-time-pcr-system.html
NDP View 2	Hamamatsu Photonics KK	https://www.hamamatsu.com/jp/ja.html

**Other**		

NMR: FT-NMR AV400NEO	Bruker	https://www.bruker.com/en/products-and-solutions/mr/nmr.html
NMR: JNM-ECZL500R	JEOL	https://www.jeol.com/products/scientific/feature_nmr/
LRMS: ACQUITY QDa	Waters	https://www.waters.com/waters/en_US/ACQUITY-QDa-Mass-Detector-for-Chromatographic-Analysis/nav.htm?cid=134761404&lset=1&locale=en_US&changedCountry=Y
UPLC-MS: BioAccord	Waters	https://www.waters.com/waters/en_US/BioAccord-LC-MS-System-for-Biopharmaceuticals/nav.htm?cid=135005818&locale=en_US
QIAcube	Qiagen	https://www.qiagen.com/jp/listpages/ez1-cards/qiacube/
Tecan i-control	Tecan	https://www.tecan.com/
NanoDrop spectrophotometer	Thermo Fisher Scientific	https://www.thermofisher.com/jp/ja/home/industrial/spectroscopy-elemental-isotope-analysis/molecular-spectroscopy/uv-vis-spectrophotometry/instruments/nanodrop.html
Acquity Premier BEH C18 1.7-μm VanGuard column	Waters	ID#186009457
7500 Real Time PCR System	Applied Biosystems	https://www.thermofisher.com/order/catalog/product/4377354?SID=srch-srp-4377354
NanoZoomer S210	Hamamatsu Photonics KK	https://www.hamamatsu.com/jp/ja.html
Mesh Nebulizer NE-U100	OMRON Corporation	https://www.healthcare.omron.co.jp/product/ne/ne-u100.html

### Experimental model and subject details

#### Cells, viruses, and test compounds

Compounds **1**–**19**, **2-TP** and NHC (EIDD-1931)^44^ were synthesized at the Faculty of Pharmaceutical Science, Tokushima University, and their chemical purity (all >95%) was verified by ultra-high-performance liquid chromatography (UPLC) and HRMS analysis (see the supplemental information). Remdesivir (GS-5734) was purchased from Merck & Co., Inc. (Rahway, NJ, USA). Molnupiravir (EIDD-2801) was purchased as LAGEVRIO Capsules^Ⓡ^ 200 mg from Merck & Co., Inc. (Rahway, NJ, USA). For *in vitro* studies, all compounds were solubilized in DMSO (Kanto Chemical Co., Inc., Tokyo, Japan). For *in vivo* studies, all compounds were dissolved in Otsuka Normal Saline (Otsuka Pharmaceutical Co., Ltd., Tokyo, Japan) or 50% DMSO for intraperitoneal administration. Vero E6 cells (ATCC: CRL-1586) were maintained in Eagle’s MEM (EMEM) containing 10% heat-inactivated fetal bovine serum (FBS) and 1% penicillin–streptomycin–glutamine (Thermo Fisher Scientific Inc., MA, USA). Vero E6 cells were inoculated with SARS-CoV-2 (SARS-CoV-2/Hu/DP/Kng/19–020; GenBank LC528232; kindly provided by Kanagawa Prefectural Institute of Public Health, Kanagawa, Japan). Two days after inoculation, culture supernatants were collected and filtered to make virus stocks.

#### Animal experiments

To assess the toxicity of C^3^Guo (**2**) in mammals, 6-week-old female C57BL/6J mice (CLEA Japan, Inc.) were given 12.5 mg/kg or 125 mg/kg of C^3^Guo (**2**) IP daily for 5 days and observed for body weight changes and clinical signs. The antiviral effects of C^3^Guo (**2**) were tested *in vivo* in a Syrian hamster model of SARS-CoV-2 infection.^38^ Male hamsters (Slc:Syrian, 5 weeks old) obtained from Japan SLC (Shizuoka, Japan) were intranasally inoculated with 1 × 10^2^ PFU of SARS-CoV-2. They were given 110 mg/kg/day of C^3^Guo (**2**) (qd) or 220 mg/kg of Molnupiravir (bid) by IP injection, starting 2 h before infection.

For daily inhalation administration, each hamster was placed in a polypropylene tube (diameter, 36 mm; length, 90 mm) connected to a nebulizer (NE-U100, Omron, Kyoto, Japan; mass median aerodynamic diameter of particles, ∼5 μm) such that only the head was exposed to aerosolized drug. A solution of 10 mg/mL of C^3^Guo (**2**), 10 mg/mL Molnupiravir, or saline was placed in the nebulizer at a volume corresponding to a 170 mg/kg dose, and hamsters were exposed until no more aerosol was produced. Approximately 0.5 mL (5 mg equivalent) remained in the nebulizer; thus, hamsters were exposed to an aerosolized volume of ∼110 mg/kg. Hamsters were observed for appearance.

Infectious viral titer in the lung was evaluated at 2 dpi (peak infection) and 5 dpi; histopathological analysis of the lungs was performed at 5 dpi after the animals were euthanized. The right lung of each hamster was weighed and homogenized with stainless steel beads (5.0 mm) in 1 mL of phosphate-buffered saline (300 rpm, 90 s) twice using a Shakeman 6 homogenizer (BMS Inc., Tokyo, Japan). After centrifugation (13,200*g*, 10 min 4°C), the supernatants were collected and used for plaque assay. The left lung was perfused with 10% phosphate-buffered formalin solution (FUJIFILM Wako Pure Chemical Corporation) for histopathological analysis. All animal experiments with SARS-CoV-2 were performed in animal biosafety level 3 (ABSL3) containment laboratories at Tokushima University. All experimental procedures were approved by the Institutional Animal Care and Use Committee (IACUC) of Tokushima University (Approval Number: T2020-116).

### Method details

#### Chemical synthesis and compound characterization

All reactions were carried out using oven-dried glassware and magnetic stirring under argon atmosphere unless otherwise stated. Analytical thin-layer chromatography (TLC) was performed on Merck Kieselgel F254 and visualized by UV light (254 nm). Column chromatography was performed using KANTO Chemical silica gel 60N (neutral). Physical data were measured as follows; nuclear magnetic resonance (NMR) spectra were recorded on FT-NMR AV400*NEO* (Bruker, MA, USA) or JNM-ECZL500R (JEOL, Kyoto, Japan). ^1^H NMR spectra were recorded at 400 or 500 MHz, referenced to in CDCl_3_ with tetramethylsilane (TMS) (0.00 ppm), DMSO-*d*_6_ (2.50 ppm), and D_2_O (4.79 ppm). ^13^C NMR spectra were recorded at 125 MHz, referenced to in CDCl_3_ with TMS (0.00 ppm). ^31^P NMR spectrum was recorded at 202 MHz, referenced to in D_2_O (0.00 ppm, phosphoric acid as an external reference). Chemical shifts are reported in parts per million (δ), and signals are expressed as s (singlet), d (doublet), t (triplet), q (quartet), m (multiplet), or br (broad). All exchangeable protons were detected by addition of D_2_O. Mass spectra were measured on a ACQUITY QDa (for LRMS, quadrupole, Waters), and BioAccord ACQUITY RDa (for HRMS, TOF, Waters). The purity of assay compounds was analyzed using UPLC-UV/MS (BioAccord ACQUITY, Waters) with an Acquity Premier BEH C18 1.7-μm VanGuard column (Waters, 50 × 2.1 mm), eluted with appropriate solvent system.

Detailed synthetic procedures and compound characterization are provided in the supplemental information, along with NMR spectra were also available.

##### Abbreviations

Standard abbreviations for the protecting groups are followed by the IUPACIUB Commission on Biochemical Nomenclature.

##### Reagents

All the reagents and solvents used were commercially available and used without further purification.

Synthesis of 3-deazadiaminopurine nucleoside (6), related to Scheme 1.

**1-[2,3,5-Tri-*O*-(*tert*-buthyldimethylsilyl)-β-D-ribofuranosyl]-5-cyanomethyl-1*H*-imidazole-4-carbonitrile (8).** To a solution of **7**^21^ (1.48 g, 2.37 mmol) in pyridine (25.0 mL) was added *p*-toluenesulfonyl chloride (TsCl) (2.71 g, 14.2 mmol), and the whole was stirred for 27 h under room temperature. The reaction mixture was quenched by the addition of ice, and the solvent was removed *in vacuo*. The residue was partitioned between AcOEt and saturated aqueous NaHCO_3_. The separated organic layer was further washed with H_2_O (twice), followed by brine, dried (Na_2_SO_4_) and concentrated *in vacuo*. The residue was purified by a silica gel column, eluted with hexane/AcOEt (9/1–2/1) to give **8** (1.40 g, 97%) as a pale brown oil. ESI-LRMS *m/z* 629 [M+Na]^+^; ESI-HRMS calcd for C_29_H_54_N_4_O_4_Si_3_ [M + H]^+^ 607.3526, found 607.3526; ^1^H NMR (CDCl_3_, 500 MHz) δ 7.79 (1 H, s, H-2), 5.67 (1 H, d, *J* = 6.7 Hz, H-1′), 4.17–4.13 (4 H, m, H-2′, H-3′ and CH_2_CN), 3.97–3.95 (1 H, H-4′, m), 3.94–3.93 (1 H, m, H-5′a), 3.80 (1 H, dd, *J* = 2.2, 11.7 Hz, H-5′b), 0.95, 0.94, and 0.82 (each s, each 9 H, *t*-Bu × 3), 0.14 (6 H, s, Me×2), 0.12, 0.11, −0.02, and −0.32 (each 3 H, each s, Me×4); ^13^C NMR (CDCl_3_, 125 MHz) δ 138.51, 128.88, 115.86, 113.90, 113.26, 89.42, 87.94, 77.33, 72.70, 63.21, 26.02, 25.76, 25.67, 18.49, 18.01, 17.84, 13.76, −4.36, −4.53, −4.60, −5.43, −5.73.

**4,6-Diamino-1-[2,3,5-tri-*O*-(*tert*-buthyldimethylsilyl)-β-D-ribofuranosyl]-1*H*-imidazo[4,5-*c*]pyridine (9).** A solution of **8** (1.40 g, 2.31 mmol) in NH_3_/MeOH (saturated at 0°C, 30.0 mL) was heated at 100°C for 12 h in a sealed stainless tube. The reaction mixture was concentrated *in vacuo*, the residue was purified by a silica gel column, eluted with MeOH in CHCl_3_ (0%–6%), to give **9** (1.11 g, 77%) as a dark red solid. ESI-LRMS *m/z* 624 [M + H]^+^; ESI-HRMS calcd for C_29_H_57_N_5_O_4_Si_3_ [M + H]^+^ 624.3791 found 624.3830; ^1^H NMR (CDCl_3_, 500 MHz) δ 7.83 (1 H, s, H-8), 5.99 (1 H, s, H-3), 5.66 (1 H, d, *J* = 7.1 Hz, H-1′), 5.23 (2 H, br s, exchangeable with D_2_O, NH_2_), 4.31 (1 H, dd, *J* = 4.5, 7.1 Hz, H-2′), 4.24 (2 H, br s, exchangeable with D_2_O, NH_2_), 4.19 (1 H, dd, *J* = 4.0, 4.5 Hz, H-3′), 4.10–4.08 (1 H, m, H-4′), 3.90 (1 H, dd, *J* = 2.9, 11.5 Hz, H-5′a), 3.81 (1 H, dd, *J* = 2.4, 11.5 Hz, H-5′b), 0.97, 0.95, and 0.78 (each 9 H, each s, *t*-Bu×3), 0.17, 0.16, 0.12, 0.11, −0.10, and −0.44 (each 3 H, each s, Me×6); ^13^C NMR (CDCl_3_, 125 MHz) δ 152.38, 149.26, 141.77, 138.43, 121.43, 88.33, 86.62, 79.24, 76.16, 72.80, 63.34, 26.07, 25.84, 25.71, 18.53, 18.09, 17.81, −4.49, −4.53, −4.65, −5.30, −5.41, −5.50.

**4,6-Diamino-1-β-D-ribofuranosyl-1*H*-imidazo[4,5-*c*]pyridine (3-deazadiaminopurine nucleoside, 6).** To a solution of **9** (250 mg, 0.4 mmol) in CH_2_Cl_2_ (5.0 mL) was added triethylamine trihydrofluoride (228 μL, 1.4 mmol) at 0°C. After being stirred at 25 h under room temperature, the solvent was removed *in vacuo*. The residue was purified by a silica gel column, eluted with MeOH in CHCl_3_ (0%–35%), to give **6** (61 mg, 54%) as a dark brown solid. The analytical and assay samples were recrystallized from MeOH–hexane. ESI-LRMS m/z 282 [M + H]^+^; ESI-HRMS calcd for [M + H]^+^ C_11_H_15_N_5_O_4_ 282.1197 found 282.1241; ^1^H NMR (DMSO-*d*_6_, 500 MHz) δ 7.91 (1 H, s, H-8), 5.78 (2 H, br s, exchangeable with D_2_O, NH_2_), 5.75 (1 H, s, H-3), 5.52 (1 H, d, *J* = 5.9 Hz, H-1′), 5.41 (1 H, d, *J* = 6.2 Hz, 2′-OH, exchangeable with D_2_O), 5.16 (1 H, d, *J* = 5.0 Hz, exchangeable with D_2_O, 3′-OH), 5.09 (2 H, br s, exchangeable with D_2_O, NH_2_), 5.00 (1 H, t, *J* = 5.3 Hz, exchangeable with D_2_O, 5′-OH), 4.29–4.24 (1 H, m H-2′), 4.06–4.02 (1 H, m, H-3′), 3.91–3.86 (1 H, m, H-4′), 3.65–3.60 (1 H, m, H-5′a), 3.58–3.53 (1 H, m, H-5′b); ^13^C NMR (DMSO-*d*_6_, 125 MHz) δ 154.60, 150.30, 141.24, 137.46, 120.16, 88.25, 85.06, 76.63, 73.35, 70.17, 61.39.

Chemical synthesis of 3-deazaguanine (C^3^Gua, 10) and 2′-deoxy-3-Deazaguanosine (dC^3^Guo, 11), related to Scheme 2.

**6-Amino-1-(2-deoxy-3,5-bis-*O*-triisopropylsilyl-β-D-ribofuranosyl)-1*H*-imidazo[4,5-*c*]pyridin-4(5*H*)-one (13).** To a solution of **12**^31^ (6.03 g, 10.1 mmol) in THF (150 mL) was added 1-(trifluoroacetyl)imidazole (1.73 mL, 15.2 mmol), and heated to reflux for 4 h. After the reaction mixture was concentrated *in vacuo*, the resulting residue was dissolved in EtOH (80.0 mL) and added to 5% aqueous Na_2_CO_3_ (40.0 mL). After being heated to reflux for 4 h, the reaction mixture concentrated *in vacuo*. The residue was partitioned between CHCl_3_ and H_2_O. The separated organic layer was further washed with H_2_O (twice) and followed by brine, dried (Na_2_SO_4_), and concentrated *in vacuo*. The residue was purified by a silica gel column, eluted with MeOH in CHCl_3_ (0%–15%), to give **13** (5.45 g, 93%) as a dark green foam. ESI-LRMS m/z 601 [M+Na]^+^; ESI-HRMS calcd for [M + H]^+^ C_29_H_54_N_4_O_4_Si_2_ 579.3756 found 579.3770; ^1^H NMR (CDCl_3_, 400 MHz) δ 12.74 (1 H, br s, exchangeable with D_2_O, NH), 7.78 (1 H, s, H-8), 6.00 (1 H, dd, *J* = 5.5, 8.3 Hz, H-1′), 5.53 (1 H, s, H-3), 4.85 (2 H, br s, exchangeable with D_2_O, NH_2_), 4.74–4.69 (1 H, m, H-3′), 4.11–4.05 (1 H, m, H-4′), 3.87 (1 H, dd, *J* = 3.3, 11.0 Hz, H-5′a), 3.81 (1 H, dd, *J* = 4.5, 11.0 Hz, H-5′b), 2.44 (1 H, ddd, *J* = 5.2, 8.3, 13.1 Hz, H-2′a), 2.37 (1 H, ddd, *J* = 2.1, 5.5, 13.1 Hz, H-2′b), 1.16–1.05 (42 H, m, TIPS); ^13^C NMR (CDCl_3_, 125 MHz) δ 158.60, 147.30, 142.92, 136.52, 124.03, 88.42, 84.89, 73.01, 72.75, 63.63, 41.64, 18.01, 18.01, 17.98, 12.08, 11.89.

**6-[{(Dimethylamino)methylene}amino]-1-(2-deoxy-3,5-bis-*O*-triisopropylsilyl-β-D-ribofuranosyl)-1*H*-imidazo[4,5-*c*]pyridin-4(5*H*)-one (14).** To a solution of **13** (3.47 g, 6.00 mmol) in DMF (60.0 mL) was added *N*,*N*-dimethylformamide dimethyl acetal (4.00 mL, 30.0 mmol), and the whole was stirred for 5.5 h at room temperature. The solvent was removed *in vacuo* and the residue was partitioned between CHCl_3_ and H_2_O. The separated organic layer was further washed with H_2_O (twice) and followed by brine, dried (Na_2_SO_4_) and concentrated *in vacuo*. The residue was purified by a silica gel column, eluted with MeOH in CHCl_3_ (0%–20%) to give **14** (3.83 g, quant) as a dark brown foam. ESI-LRMS m/z 656 [M+Na]^+^; ESI-HRMS calcd for C_32_H_59_N_5_O_4_Si_2_ 634.4178 found 634.4184; ^1^H NMR (CDCl_3_, 400 MHz) δ 8.91 (1 H, br s, exchangeable with D_2_O, NH), 7.85 (1 H, s, H-8), 7.82 (1 H, s, CH), 6.06 (1 H, dd, *J* = 5.5, 8.3 Hz, H-1′), 5.77 (1 H, s, H-3), 4.75–4.71 (1 H, m, H-3′), 4.12–4.07 (1 H, m, H-4′), 3.86 (1 H, dd, *J* = 3.4, 11.0 Hz, H-5′a), 3.79 (1 H, dd, *J* = 4.8, 11.0 Hz, H-5′b), 3.12 and 3.04 (each 3 H, each s, Me×2), 2.50 (1 H, ddd, *J* = 5.2, 8.3, 13.1 Hz, H-2′a), 2.40 (1 H, ddd, *J* = 2.2, 5.5, 13.1 Hz, H-2′b), 1.21–1.04 (42 H, m, TIPS); ^13^C NMR (CDCl_3_, 125 MHz) δ 158.21, 154.32, 150.51, 141.43, 136.87, 127.83, 88.57, 84.83, 78.28, 72.80, 63.67, 41.61, 40.66, 34.74, 18.01, 17.96, 12.08, 11.87.

**6-[{(Dimethylamino)methylene}amino]-1-(2-deoxy-β-D-ribofuranosyl)-1*H*-imidazo[4,5-*c*]pyridin-4(5*H*)-one (15).** To a solution of **14** (3.73 g, 5.88 mmol) in THF (60.0 mL) was added TBAF (1.0 M THF solution, 14.7 mL, 14.7 mmol) at 0°C. After being stirred for 50 min at room temperature, the reaction mixture was concentrated *in vacuo*. The residue was crystallized from MeOH to give **15** (1.56 g, 83%) as white crystals. ESI-LRMS m/z 344 [M+Na]^+^; ESI-HRMS calcd for [M + H]^+^ C_14_H_19_N_5_O_4_ 322.1510 found 322.1529; ^1^H NMR (DMSO-*d*_6_, 400 MHz) δ 10.59 (1 H, br s, exchangeable with D_2_O, NH), 8.04 (1 H, s, H-8), 8.00 (1 H, s, CH), 6.10 (1 H, dd, *J* = 6.0, 7.3 Hz, H-1′), 6.07 (1 H, s, H-3), 5.28 (1 H, d, *J* = 3.8 Hz, exchangeable with D_2_O, 3′-OH), 4.99 (1 H, t, *J* = 5.2 Hz, exchangeable with D_2_O, 5′-OH), 4.39–4.32 (1 H, m, H-3′), 3.87–3.81 (1 H, m, H-4′), 3.59–3.48 (2 H, m, H-5′a and H-5′b), 3.06 (3 H, s, CH_3_), 2.94 (3 H, s, CH_3_), 2.48–2.44 (1 H, m, H-2′a), 2.25 (1 H, ddd, H-2’b, *J* = 3.1, 6.0, 13.4 Hz); ^13^C NMR (DMSO-*d*_6_, 125 MHz) δ 157.38, 155.05, 150.99, 140.94, 137.62, 126.92, 87.65, 84.28, 77.05, 70.52, 61.51, 34.15.

**6-[{(Dimethylamino)methylene}amino]-1*H*-imidazo[4,5-*c*]pyridin-4(5*H*)-one (16).** A solution of **15** (1.56 g, 4.85 mmol) in 1.0 N HCl (50 mL) was stirred at 50°C for 4 h. After being cooled to room temperature, the reaction mixture was neutralized by the addition of 1.0 N NaOH at 0°C, and the solvent was removed *in vacuo*. The residue was purified by a silica gel column, eluted with MeOH in CHCl_3_ (0%–30%) to give **16** (498 mg, 50%) as a pale pink solid. ESI-LRMS m/z 206 [M + H]^+^; ESI-HRMS calcd for [M + H]^+^ C_9_H_11_N_5_O 206.1036 found 206.1036; ^1^H NMR (DMSO-*d*_6_, 400 MHz) δ 12.90 (0.4 H, br s, exchangeable with D_2_O, NH), 12.24 (0.6 H, br s, exchangeable with D_2_O, NH), 10.66 (0.4 H, br s, exchangeable with D_2_O, NH), 10.43 (0.6 H, br s, exchangeable with D_2_O, NH), 7.99–7.74 (2 H, m, H-8 and CH), 5.94 (0.4 H, s, H-3), 5.79 (0.6 H, s, H-3), 3.04 and 2.92 (each 3 H, each s, Me×2).

**6-Amino-1*H*-Imidazo[4,5-*c*]pyridin-4(5*H*)-one (C**^**3**^**Gua, 10).**^29^ A solution of **16** (74.0 mg, 0.36 mmol) in NH_3_/MeOH–H_2_O (3:1) (saturated at 0°C, 8.0 mL) was stirred at room temperature for 43 h. The solvent was removed *in vacuo*. The resulting residue containing **10** was diluted in potassium phosphate buffer (1.0 mL, c = 10 mmol/L, pH 8.0) and purified on a C18 cartridge column (YMC Dispo SPE C18), eluted with 5% MeOH to give C^3^Gua (**10**) (41.9 mg, 77%) as a pale pink solid. ESI-LRMS m/z 173 [M+Na]^+^; ESI-HRMS calcd for [M + H]^+^ C_6_H_6_N_4_O 151.0614 found 151.0648; ^1^H NMR (DMSO-*d*_6_, 400 MHz) δ 12.68 (0.2 H, br s, exchangeable with D_2_O, NH), 11.89 (0.8 H, br s, exchangeable with D_2_O, NH), 10.36 (0.2 H, br s, exchangeable with D_2_O, NH), 10.15 (0.8 H, br s, exchangeable with D_2_O, NH), 7.85 (0.2 H, s, H-8), 7.61 (0.8 H, s, H-8), 5.52–5.18 (2 H, m, exchangeable with D_2_O, NH_2_), 5.52–5.18 (1 H, m, H-3).

**6-Amino-1-(2-deoxy-β-D-ribofuranosyl)-1*H*-imidazo[4,5-*c*]pyridin-4(5*H*)-one (dC**^**3**^**Guo, 11).**^30^ A solution of **15** (104 mg, 0.32 mmol) in 28% NH_4_OH (5.0 mL) was allowed to stand at room temperature for 7 days. The reaction mixture was concentrated *in vacuo*, the resulting solid was crystallized from MeOH–hexane to give **11** (21 mg, 24%) as white crystals. ESI-LRMS *m/z* 267 [M + H]^+^; ^1^H NMR (CDCl_3_, 500 MHz) δ 10.28 (1 H, s, exchangeable with D_2_O, NH), 7.86 (1 H, s, H-8), 5.93 (1 H, t, *J* = 6.9 Hz, H-1′), 5.56 (2 H, br s, exchangeable with D_2_O, NH_2_), 5.43 (1 H, s, H-3), 5.31 (1 H, d, *J* = 4.3 Hz, exchangeable with D_2_O, 3′-OH), 4.90 (1 H, t, *J* = 5.4 Hz, exchangeable with D_2_O, 5′-OH), 4.33–4.26 (1 H, m, H-3′), 3.83–3.76 (1 H, m, H-4′), 3.55–3.34 (2 H, m, H-5′a, H-5′b), 2.48–2.41 (1 H, m, H-2′a), 2.21 (1 H, ddd, *J* = 3.3, 6.9, 13.4 Hz, H-2′b).

Synthesis of 3-Deazaguanosine 5′-triphosphate (2-TP), related to Scheme 3.

**6-[{(Dimethylamino)methylene}amino]-1-(5-*O*-*tert*-butyldimethylsilyl-2,3-*O*-isopropylidene-β-D-ribofuranosyl)-1*H*-imidazo[4,5-*c*]pyridin-4(5*H*)-one (18).** To a solution of **17**^22^ (273 mg, 0.60 mmol) in DMF (6.0 mL) was added *N*,*N*-dimethylformamide dimethyl acetal (358 μL, 3.0 mmol), and the whole was stirred for 4 h at room temperature. The solvent was removed *in vacuo* and the residue was partitioned between CHCl_3_ and H_2_O. The separated organic layer was further washed with H_2_O (twice), followed by brine. The separated organic layer was dried (Na_2_SO_4_) and concentrated *in vacuo*. The residue was purified by a silica gel column, eluted with MeOH in CHCl_3_ (0%–10%), to give **18** (288 mg, 98%) as a white foam. ESI-LRMS *m/z* 492 [M + H]^+^; ESI-HRMS calcd for [M + H]^+^ C_23_H_37_N_5_O_5_Si [M + H]^+^ 492.2637, found 492.2641; ^1^H NMR (CDCl_3_, 500 MHz) δ 9.05 (1 H, br s, exchangeable with D_2_O, NH), 7.86 (1 H, s, H-8), 7.81 (1 H, s, CH), 5.81 (1 H, d, *J* = 2.9 Hz, H-1′), 5.78 (1 H, s, H-3), 4.87 (1 H, dd, *J* = 2.9, 6.2 Hz, H-2′), 4.84 (1 H, dd, *J* = 2.9, 6.2 Hz, H-3′), 4.42–4.39 (1 H, m, H-4′), 3.85 (1 H, dd, *J* = 3.3, 11.2 Hz, H-5′a), 3.79 (1 H, dd, *J* = 3.3, 11.2 Hz, H-5′b), 3.12 and 3.04 (each 3 H, each s, NMe×2), 1.62 and 1.39 (each 3 H, each s, Me×2), 0.85 (9 H, s, *t*-Bu), 0.05 and 0.04 (each 3 H, each s, SiMe×2); ^13^C NMR (CDCl_3_, 125 MHz) δ 157.91, 154.25, 150.45, 140.50, 136.99, 128.03, 114.32, 92.38, 86.56, 85.46, 81.21, 77.79, 63.35, 40.69, 34.78, 27.32, 25.89, 25.35, 18.34, −5.41, −5.61.

**6-[{(Dimethylamino)methylene}amino]-1-(2,3-*O*-isopropylidene-β-D-ribofuranosyl)-1*H*-imidazo[4,5-*c*]pyridin-4(5*H*)-one (19).** To a solution of **18** (263 mg, 0.53 mmol) in THF (11.0 mL) was added 1M TBAF in THF (640 μL, 0.64 mmol) at 0°C, and the whole was stirred for 4 h at room temperature. The solvent was removed *in vacuo*. The residue was purified by a silica gel column, eluted with MeOH in CHCl_3_ (0%–25%), to give **19** (187 mg, 93%) as a white solid. ESI-LRMS *m/z* 400 [M+Na]^+^; ESI-HRMS calcd for C_17_H_23_N_5_O_5_ [M + H]^+^ 378.1772, found 378.1788; ^1^H NMR (DMSO-*d*_6_, 500 MHz) δ 10.64 (1 H, br s, exchangeable with D_2_O), 8.04 (1 H, s, H-8), 7.97 (1 H, s, CH), 6.04 (1 H, s, H-3), 5.90 (1 H, d, *J* = 3.4 Hz, H-1′), 5.14–5.12 (1 H, m, H-2′), 5.14–5.12 (1 H, m, exchangeable with D_2_O, OH), 4.93 (1 H, dd, *J* = 2.8, 6.3 Hz, H-3′), 4.16–4.13 (1 H, m, H-4′), 3.54–3.45 (2 H, m, H-5′a, H-5′b), 3.06 and 2.91 (each 3 H, each s, NMe×2), 1.55 and 1.33 (each 3 H, Me×2); ^13^C NMR (DMSO-*d*_6_, 125 MHz) δ 157.37, 155.12, 151.15, 140.95, 137.83, 127.02, 113.56, 89.83, 85.53, 83.26, 80.76, 77.39, 61.13, 34.17, 27.00, 25.21, 13.55.

**6-Amino-1-(β-D-ribofuranosyl)-1*H*-imidazo[4,5-*c*]pyridin-4(5*H*)-one 5′**-**triphosphate (2-TP).** To a solution of **19** (150 mg, 0.4 mmol) in pyridine (0.4 mL) and 1,4-dioxane (1.4 mL) was added an 1.0 M solution of 2-chloro-4*H*-1,2,3-dioxaphosphorin-4-one in 1,4-dioxane (0.44 μL, 0.44 mmol). After 15 min, a 0.5 M solution of bis(tri-*n*-butylammonium)pyrophosphate in DMF (1.2 mL, 0.6 mmol) and tri-*n*-butylamine (0.40 mL, 1.7 mmol) were quickly added, and the reaction mixture was stirred for 10 min. A solution of 1% iodine in pyridine/water (98/2, v/v) (ca. 8 mL) was then added. After 5 min, the excess iodine was decomposed by adding 5% aqueous solution of Na_2_S_2_O_3_ (ca. 5 mL), and the reaction mixture was stirred for 5 min. The reaction mixture was concentrated *in vacuo*, and ammonium hydroxide (28%, 5 mL) was added to the residue. After 20 h, the solution was concentrated *in vacuo*, and the residue was added 50% trifluoro acetic acid (TFA) (10 mL) at 0°C, and the whole was stirred for 2 h at room temperature. The solvent was removed *in vacuo* and co-evaporated with EtOH–H_2_O (1:1), and the resulting residue was diluted in water (300 mL). The solution was applied to a DEAE Sephadex column (2.1 × 20 cm), which was eluted with a linear gradient of 800 mL each of water and 0.8 M triethylammonium bicarbonate (TEAB) buffer. Fractions containing **2-TP** were concentrated *in vacuo*, and the residue was co-evaporated with EtOH. The residue was dissolved in water (5 mL), which was applied to a column of DIAION PK 212 (H^*+*^ form) and then DIAION WK 20 (Na^+^ form), and the fractions containing the desired triphosphates were concentrated *in vacuo* to give **2-TP** as a tri-sodium salt (47 mg, 20%) as a brown solid. ESI-LRMS *m/z* 520 [M–H]^–^; ESI-HRMS calcd for C_11_H_17_N_4_O_14_P_3_ [M–H]^–^ 520.9881, found 520.9908; ^1^H-NMR (D_2_O, 500 MHz) δ 7.95 (1 H, s, H-8), 5.75 (1 H, d, *J* = 7.5 Hz, H-1′), 4.60 (1 H, dd, *J* = 5.6, 7.5 Hz, H-2′), 4.53 (1 H, dd, *J* = 2.4, 5.6 Hz, H-3′), 4.29–4.26 (2 H, m, H-4′, H-5′a), 4.15–4.11 (1 H, m, H-5′b); ^31^P-NMR (D_2_O, 202 MHz) δ −5.08 (d, *J* = 20 Hz), −10.17 (d, *J* = 20 Hz), −20.84 (t, *J* = 20 Hz).

#### Antiviral assays *in vitro*

Vero E6 cells were seeded at a density of 3×10^4^ cells per well in a 96-well plate. On the next day, culture media were replaced with 2% FBS-EMEM containing the indicated concentration of the compounds and preincubated at 37°C for 1 h. SARS-CoV-2 was then added at a multiplicity of infection (MOI) of 0.001, and the cells were incubated again at 37°C for 1 h. Virus-containing medium was then replaced with the compound-containing medium and the cultures were incubated at 37°C for 3 days. On day 3 post-infection, culture supernatants were collected to measure viral copy number, and the cells in the wells were fixed by 10% formalin solution (FUJIFILM Wako Pure Chemical Corporation, Osaka, Japan) containing 0.5% crystal violet (FUJIFILM Wako Pure Chemical Corporation) to assess cytopathic effects (CPE assay). Viral titer was determined by the plaque assay method as described previously.^47^ All *in vitro* experiments handling SARS-CoV-2 were conducted at biosafety level 3 (BSL3) in the Tokushima University.

#### Quantification of SARS-CoV-2 viral RNA genome by qRT-PCR

Viral RNA in the collected supernatants was automatically extracted by using a QIAamp Viral RNA Mini kit (Qiagen, Hilden, Germany) and QIAcube system (Qiagen). The copy number of each viral RNA sample was measured using a primer pair specific for the SARS-CoV-2 N gene [5′-d(AAATTTTGGGGGACCAGGAAC)-3′ and 5′-d(TGGCACCTGTGTAGGGTCAAC)-3′]^45^ and a Fast SYBR Green RT-PCR kit (Qiagen) in accordance with the manufacturer’s instructions.

#### SARS-CoV-2 RdRp-dependent RNA synthesis assay

Recombinant SARS-CoV-2 RdRp complex (RDRP-100S2) and an RNA polymerase assay kit (S2RPA100K) were purchased from ProFoldin (Hudson, MA, USA). RNA synthesis assays were performed in a 50-μL volume with or without 10 μM **2-TP** or ddGTP (BIOLOG Life Science Institute GmbH & Co. KG, Bremen, Germany) in accordance with the manufacturer’s instructions. Reactions were incubated at 34°C for 2 h and then stopped by adding fluorescence dye (150 μL). Fluorescence intensity (Ex = 485 nm, Em = 535 nm) was measured by using a plate reader (Tecan i-control, Tecan, Männedorf, Switzerland).

#### Capping analysis

The VCE capping reaction was performed as reported by Ohno et al.^46^ To evaluate RNA capping efficiency, a short RNA [5′-r(gggcgaauuaa)-3′] with 5′-triphosphate was prepared by *in vitro* transcription. For the template, two oligo DNAs [5′-d(CAGTGAATTGTAATACGACTCACTATAGGGC)-3′, and 5′-d(TTAATTCGCCCTATAGTGAGTCGTATTACAATTCACTG)-3′] were annealed by heating and cooling in hybridization buffer (10 mM Tris–Cl, 100 mM NaCl, pH 7.6). Using the template and MEGAshortscript (Thermo Fisher Scientific), transcription was carried at 42°C overnight. After treatment with DNase, the transcript was purified by denaturing PAGE (8.0 M urea, 0.5×TBE buffer, 20% polyacrylamide gel). Eluted RNA was extracted with phenol/chloroform and precipitated with ethanol. The concentration of recovered RNA was measured by using a NanoDrop spectrophotometer (Thermo Fisher Scientific). Capping reactions were then carried out by using the ScriptCap m^7^G Capping System (C-SCCS2250, CellScript, WI, USA) at a scale of 1/5 (20 μL) in accordance with the manufacturer’s protocol. For 200 pmol of RNA, 20 nmol of GTP or **2-TP** and 8 units of Script-Cap Capping Enzyme (VCE) were incubated in ScriptCap Capping Buffer with 0.1 mM SAM and ScriptGuard RNase Inhibitor at 37°C. After 2 h, an aliquot of sample was analyzed by UPLC-MS (BioAccord, Waters) using an Acquity Premier BEH C18 1.7-μm VanGuard column (Waters, 50 × 2.1 mm), eluted with linear gradient of 2.5%–25% MeOH containing 20 mM Et_3_N and 40 mM 1,1,1,3,3,3-hexafluoropropan-2-ol at a flow rate of 0.3 mL/min for 20 min.

#### Histopathology

Lung tissues were fixed in 10% phosphate-buffered formalin (FUJIFILM Wako Pure Chemical Corporation) and routinely embedded in paraffin, sectioned, and stained with hematoxylin and eosin (HE), The slides were scanned by NanoZoomer S210 (Hamamatsu Photonics KK, Shizuoka, Japan), and the degree of pneumonia was assessed by measuring the percentage area of inflammation within the total area of each section using NDP View 2 software (Hamamatsu Photonics KK).

### Quantification and statistical analysis

Prism version 9.5.1 (GraphPad, MA, USA) was used for data analysis. The data in bar graphs are presented as mean +SEM or ±SEM. One-way ANOVA followed by Dunnett’s multiple comparisons test was performed to determine statistical significance among groups. A *p* value of <0.05 was considered statistically significant: ^∗^p < 0.05, ^∗∗^p < 0.01, ^∗∗∗^p < 0.001, and ^∗∗∗∗^p < 0.0001.
