# Supplementary material for: 3-Deazaguanosine inhibits SARS-CoV-2 viral replication and reduces the risk of COVID-19 pneumonia in hamster
Source: iScience. 2025 Mar 1;28(4):112140. doi: 10.1016/j.isci.2025.112140 (PMC11960675; doi:10.1016/j.isci.2025.112140)
Supplement: Document S1. Figures S1–S5 and Data S1–S30 [file mmc1.pdf]

iScience, Volume *28*

Supplemental information

3-Deazaguanosine inhibits SARS-CoV-2 viral replication and reduces the risk of COVID-19 pneumonia in hamster

Noriko Saito-Tarashima, Takaaki Koma, Naoto Hinotani, Keigo Yoshida, Moka Ogasa, Akiho Murai, Syuya Inoue, Tomoyuki Kondo, Naoya Doi, Koichi Tsuneyama, Masako Nomaguchi, and Noriaki Minakawa

Figure S1: Evaluation of anti-SARS-CoV-2 activity of 3-deazapurine ribonucleosides 1–3 by CPE assay, related to Figure 2. Mock, an uninfected control; DMSO, an infected control without any compound.

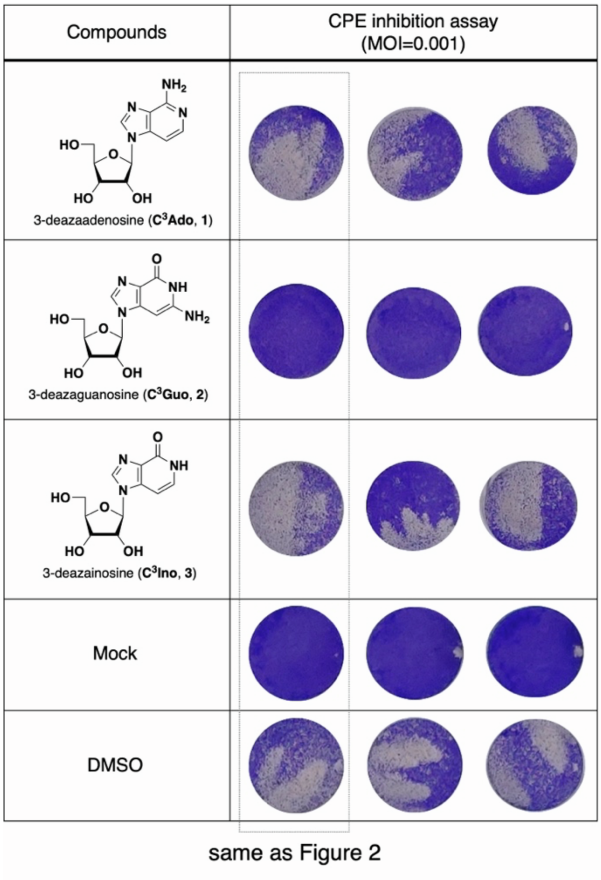

Figure S2: Dose–dependent inhibition of SARS-CoV-2 by C³Guo (2), related to Figure 2. Data from three independent experiments are presented as mean + SEM (N=6). Statistically significant differences between treated and untreated groups were determined by one-way ANOVA followed by Dunnett's multiple comparisons test: * $p \leq 0.05$, and *** $p \leq 0.001$.

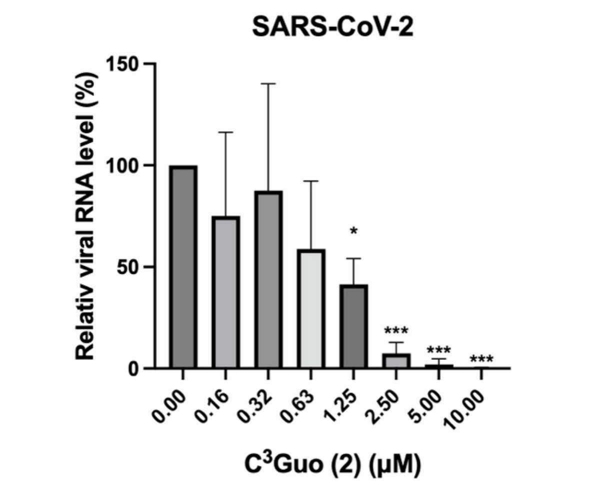

Figure S3: Predicted activation mechanism of C³Guo (2), related to Table 1.

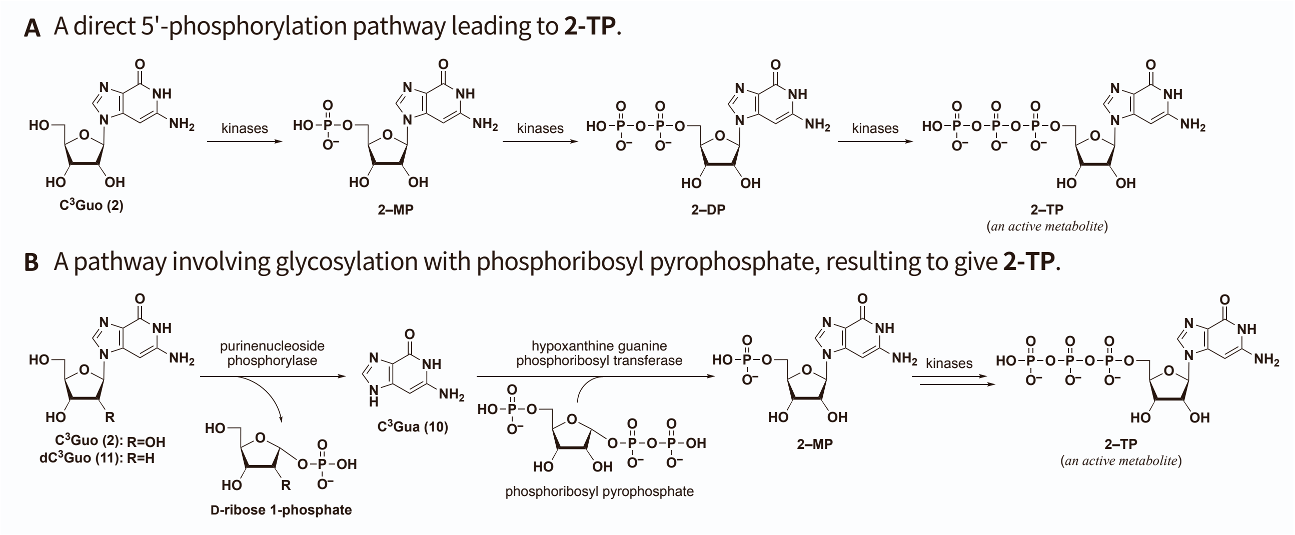

Figure S4: LC-MS analysis of the VCE capping reaction in the presence of 2-TP, related to Figure 3.

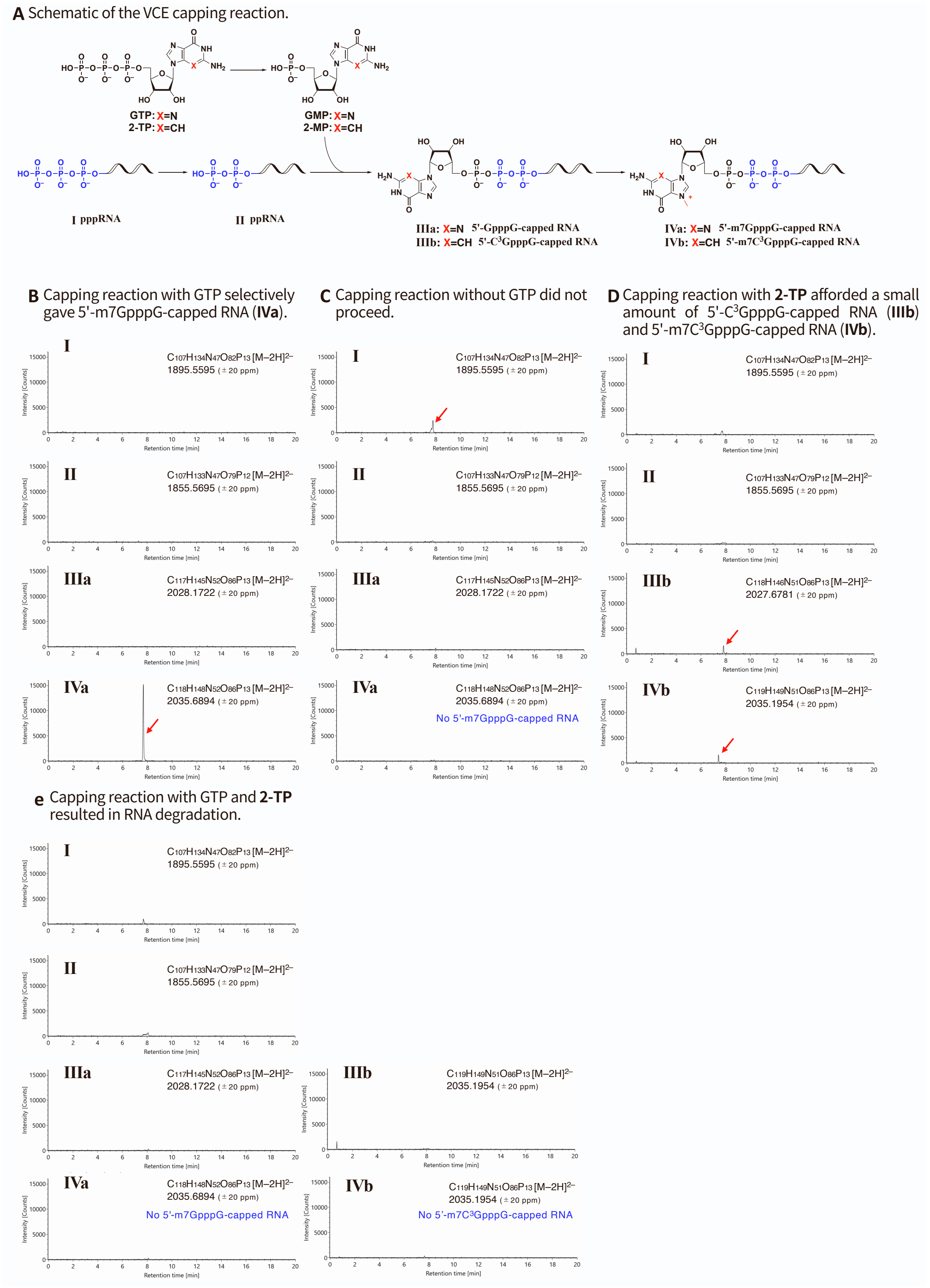

Figure S5: Body weight changes in C57BL/6J mice during repeated dosing, related to Figure 4. Data are presented as mean ± SEM [C^{3}Guo (**2**), each *n* = 2; Vehicle, *n* = 4].

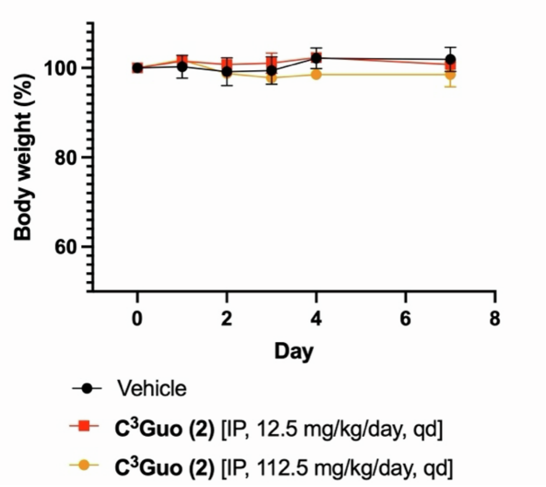

Data S1: ^{1}H NMR spectra 500 MHz, $CDCl_3$) of compound 8, related to STAR method.

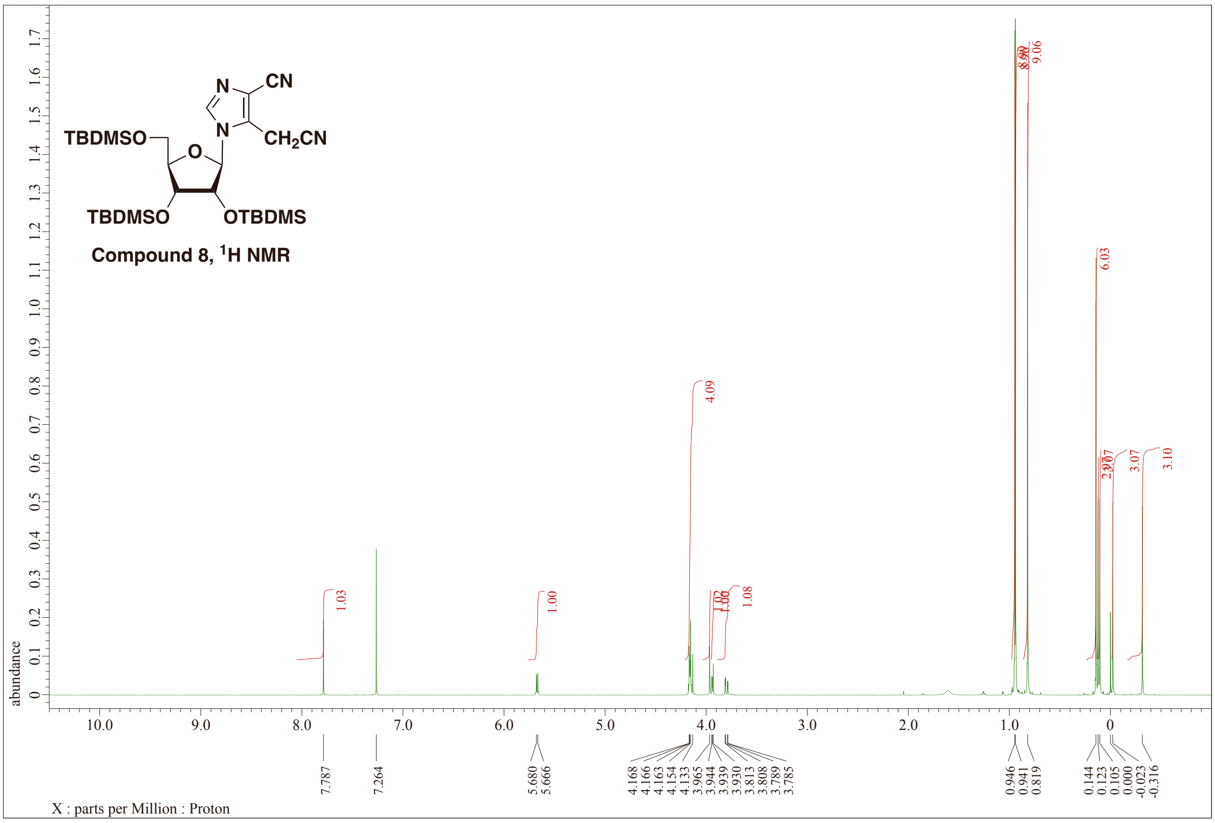

Data S2: ^{13}C NMR spectra (125 MHz, $CDCl_3$) of compound 8, related to STAR method.

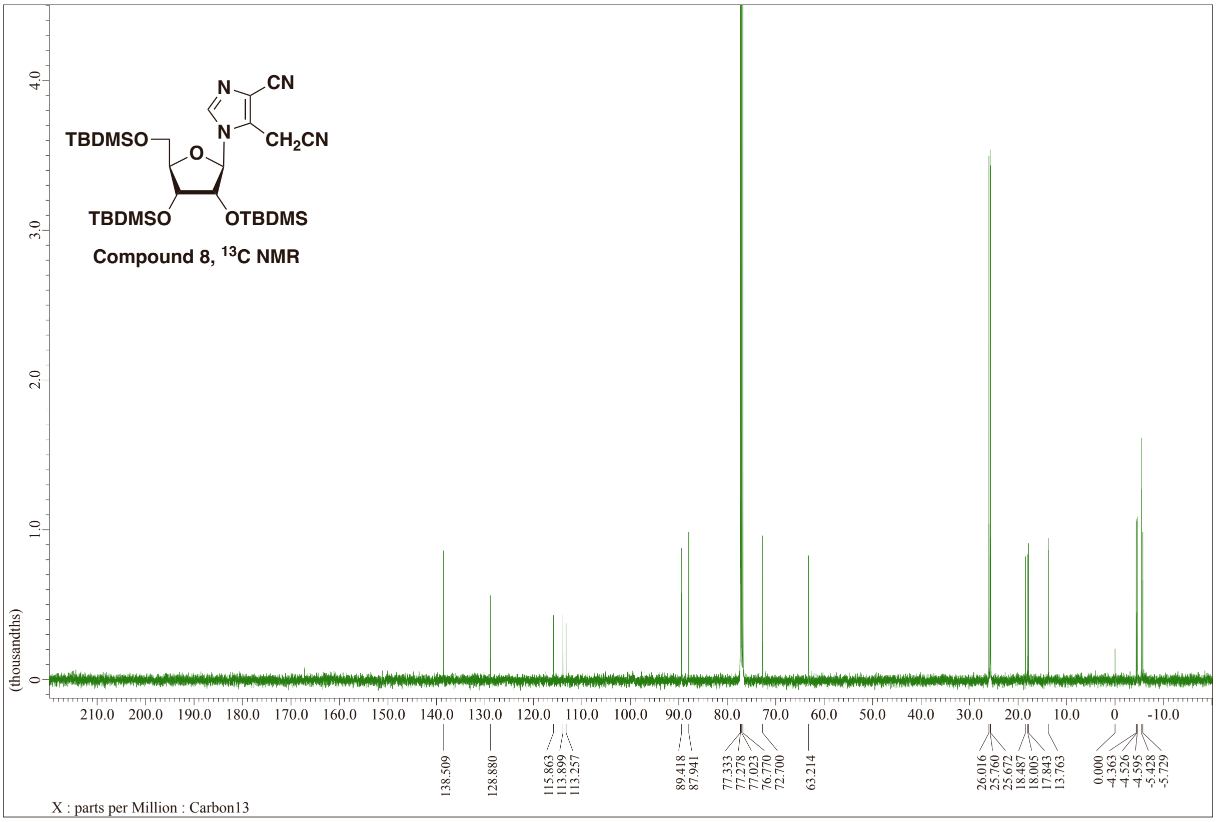

Data S3: ^{1}H NMR spectra (500 MHz, $CDCl_3$) of compound 9, related to STAR method.

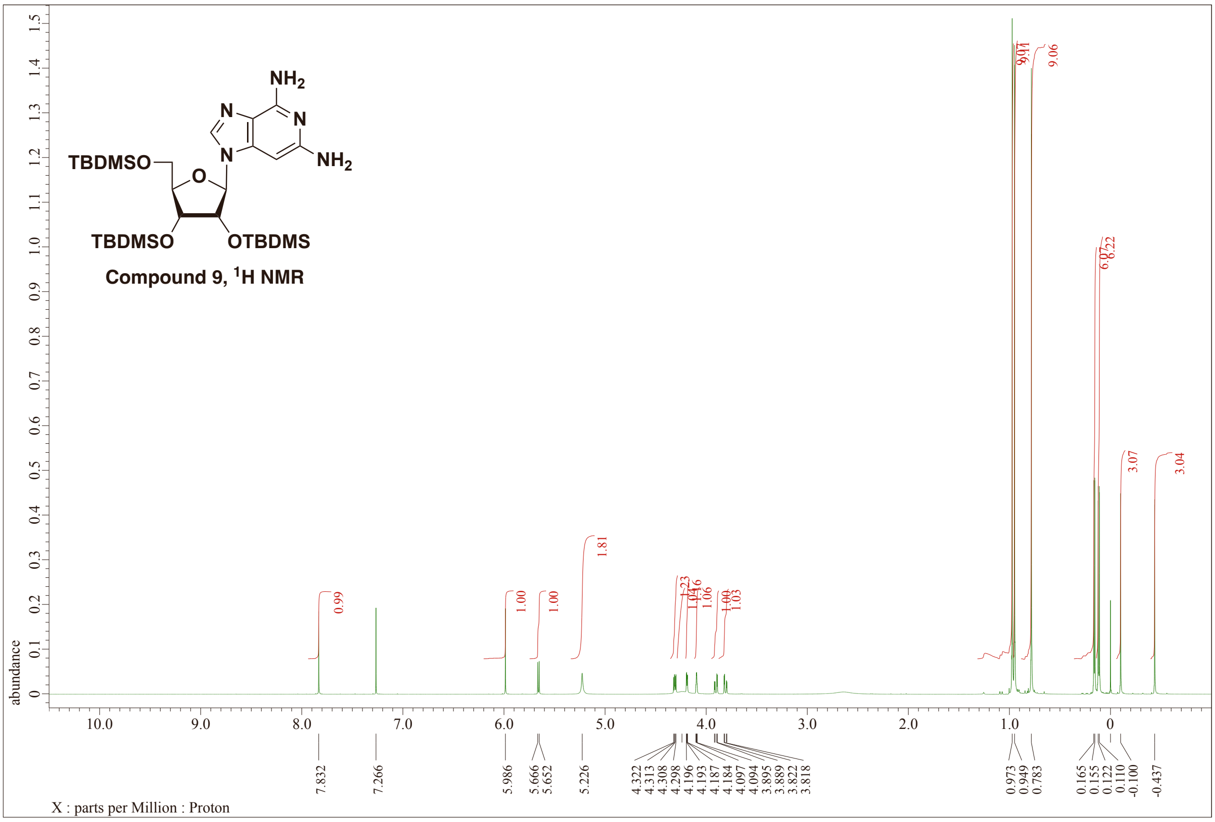

Data S4: ^{13}C NMR spectra (125 MHz, $CDCl_3$) of compound 9, related to STAR method.

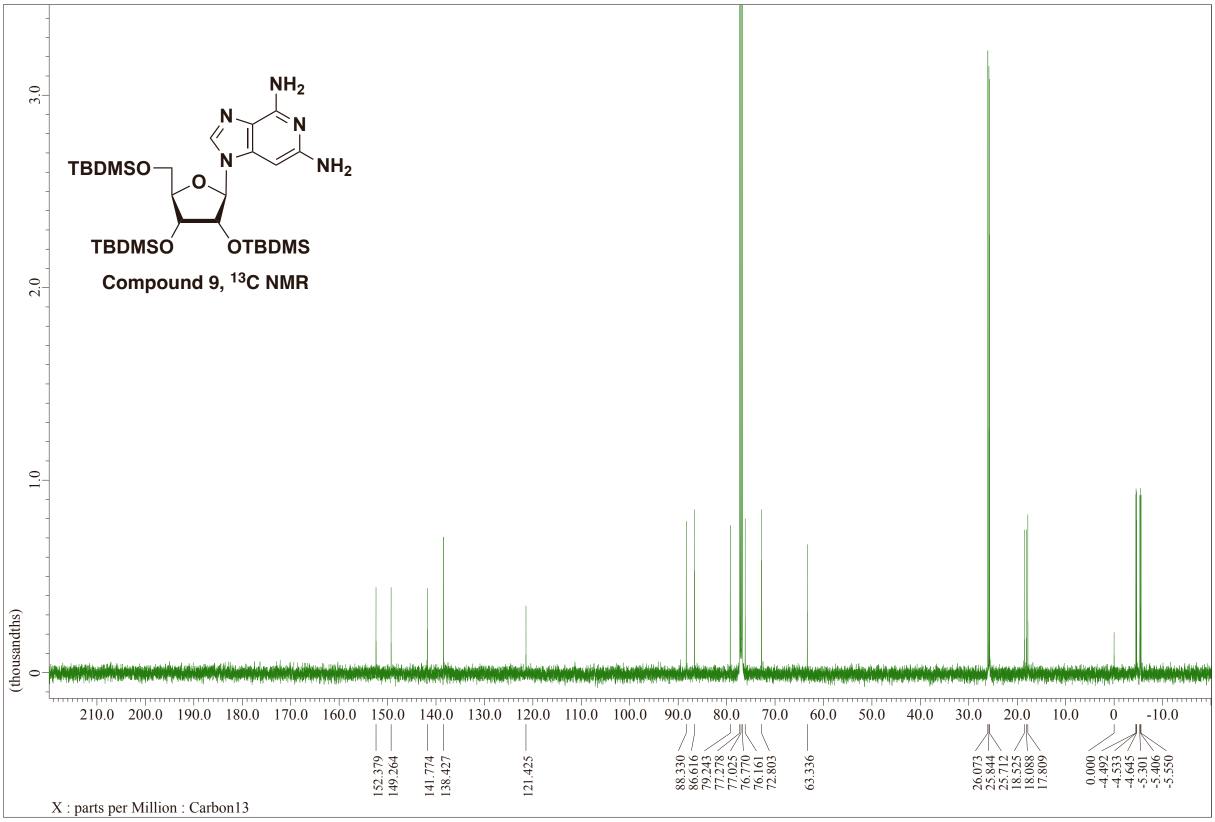

Data S5: ^{1}H NMR spectra (500 MHz, DMSO-d_6) of compound 6, related to STAR method.

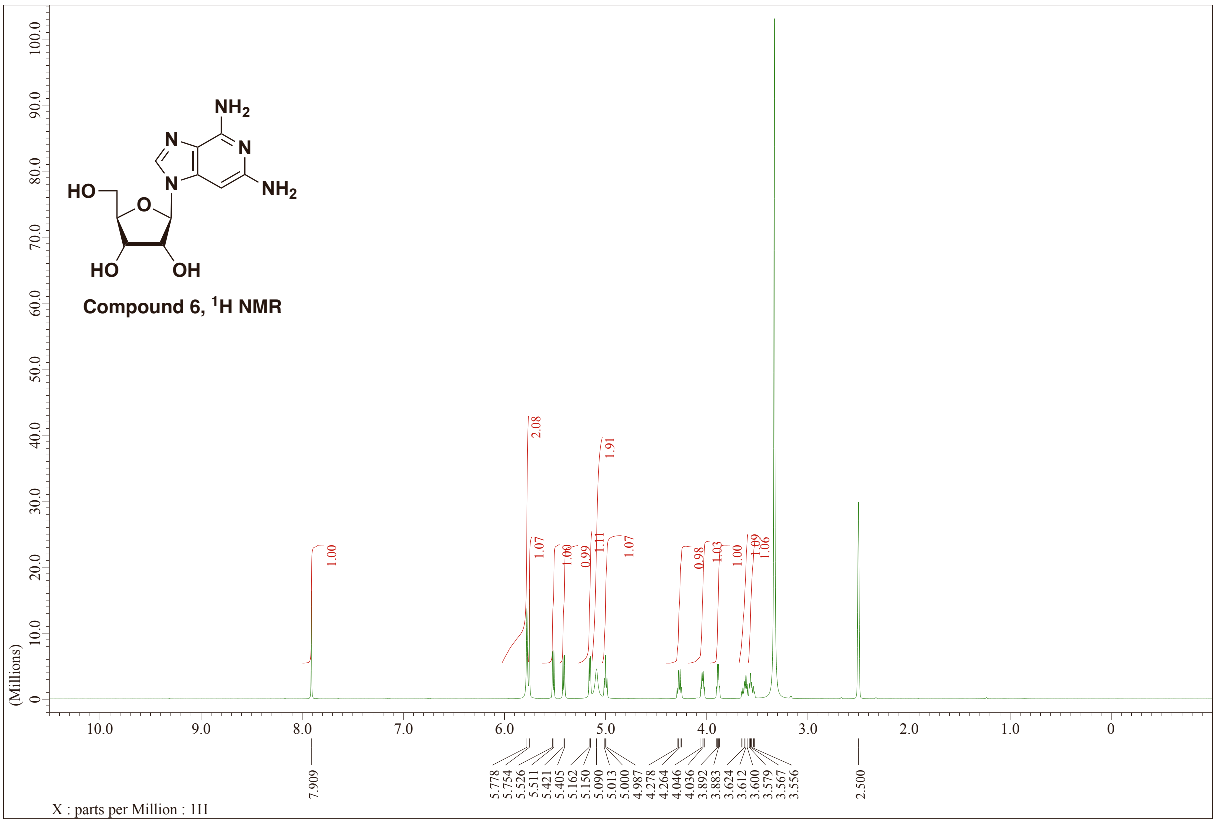

Data S6: ^{13}C NMR spectra (125 MHz, DMSO-d_6) of compound 6, related to STAR method.

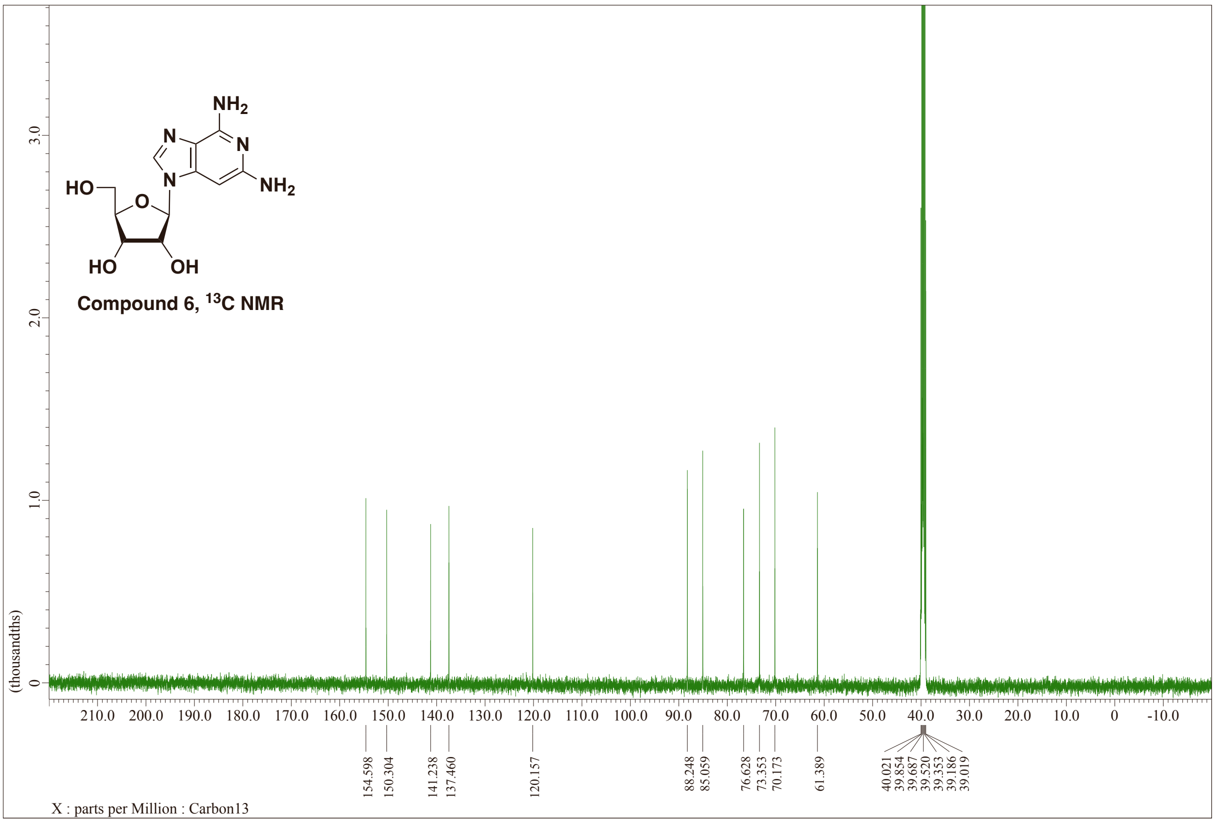

Data S7: ^{1}H NMR spectra (400 MHz, $CDCl_3$) of compound 13, related to STAR method.

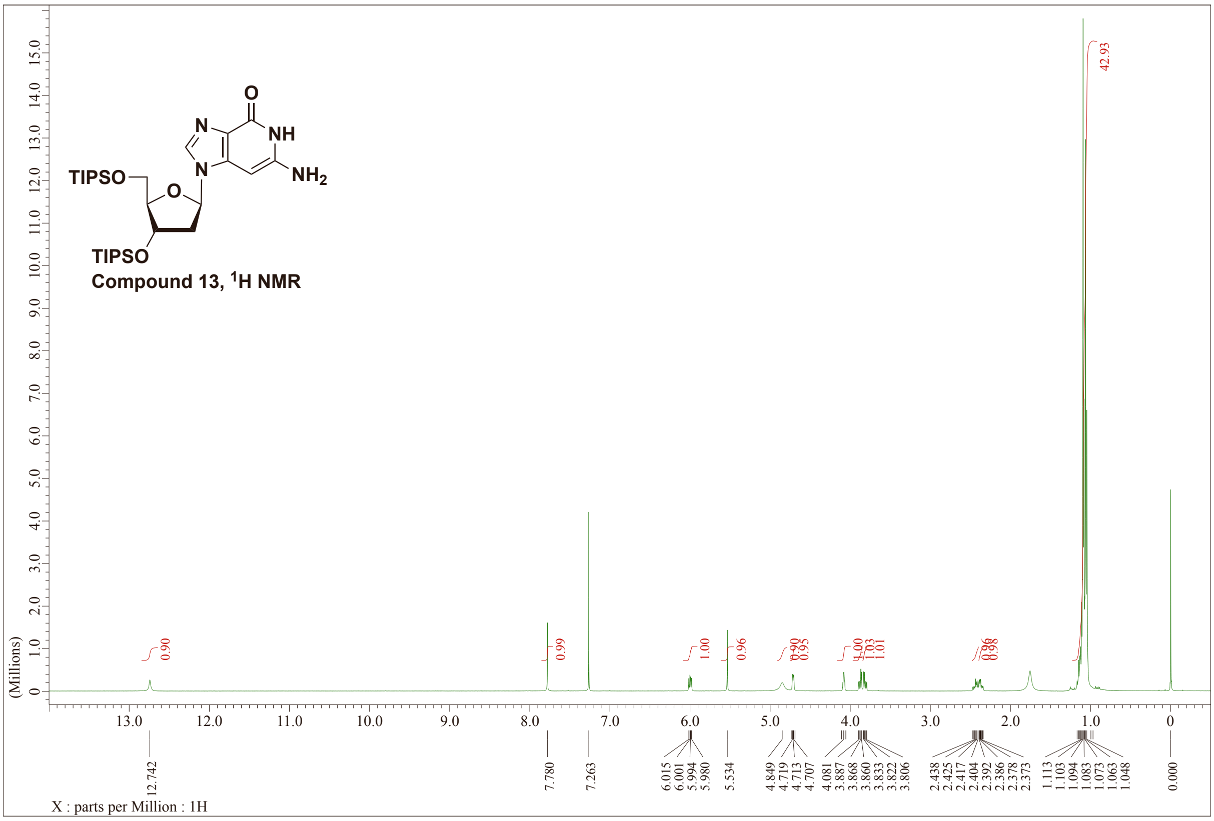

Data S8: ^{13}C NMR spectra (125 MHz, $CDCl_3$) of compound 13, related to STAR method.

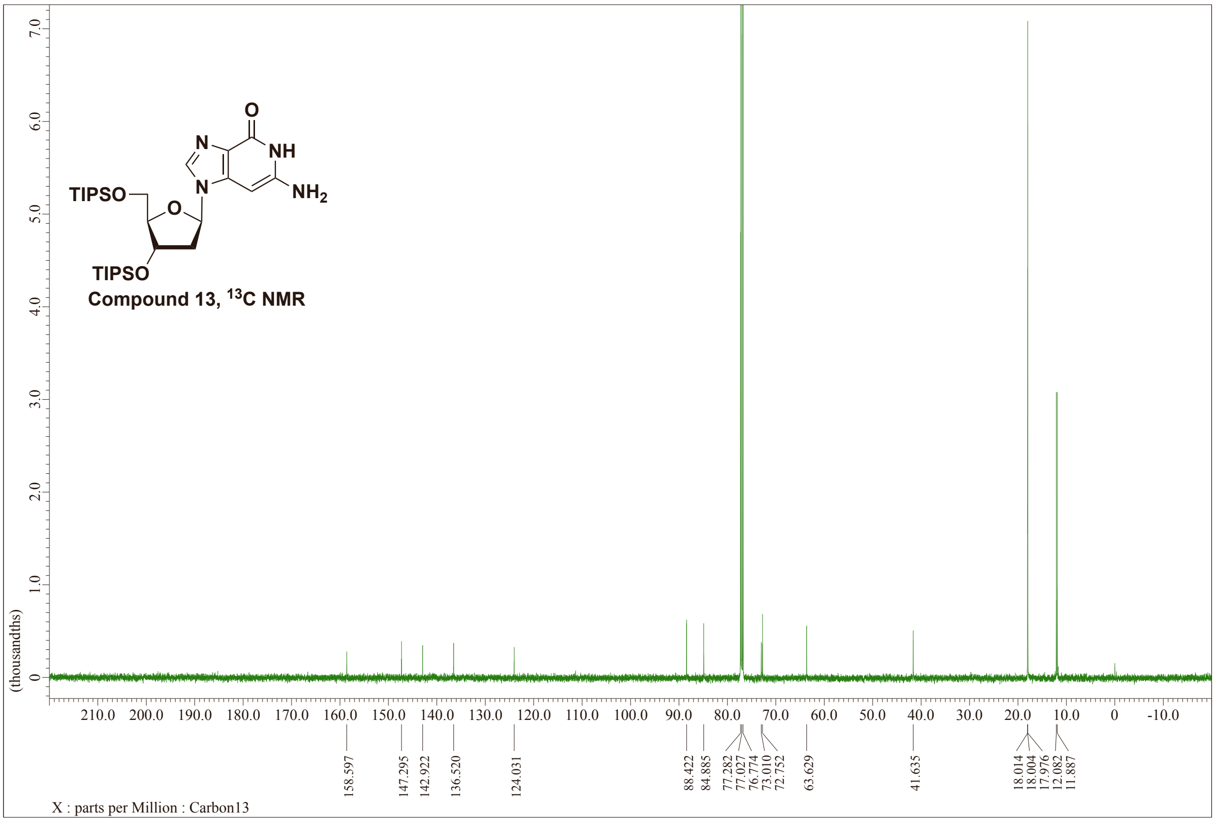

Data S9: ^{1}H NMR spectra (400 MHz, $CDCl_3$) of compound 14, related to STAR method.

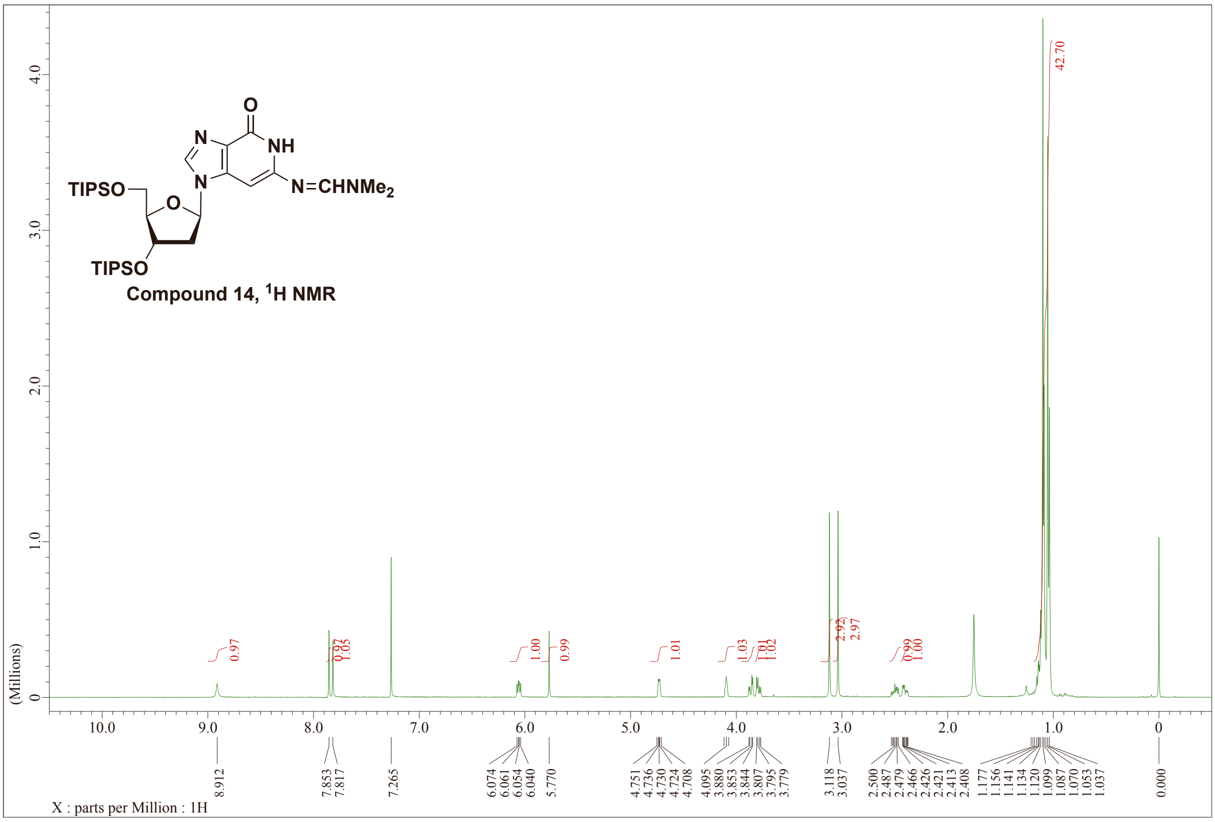

Data S10: ^{13}C NMR spectra (125 MHz, $CDCl_3$) of compound 14, related to STAR method.

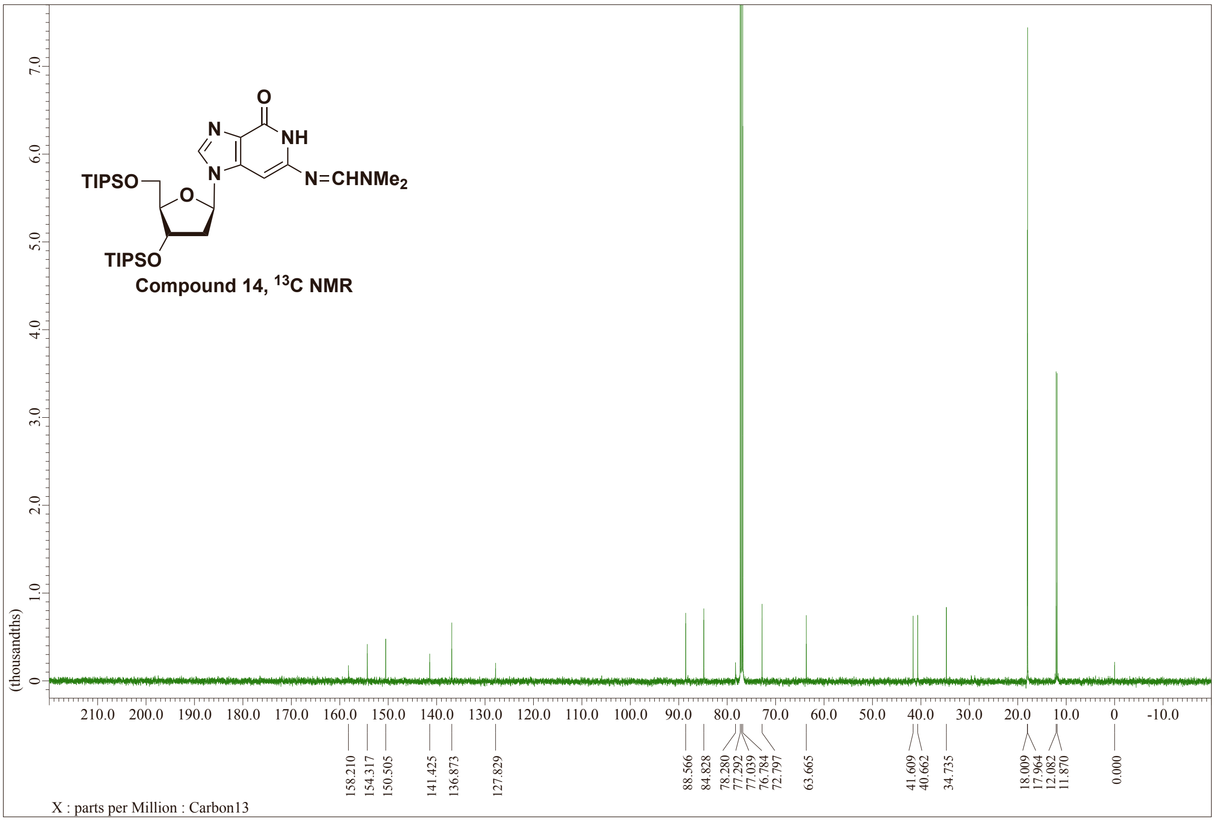

Data S11: ^{1}H NMR spectra (400 MHz, DMSO-d_6) of compound 15, related to STAR method.

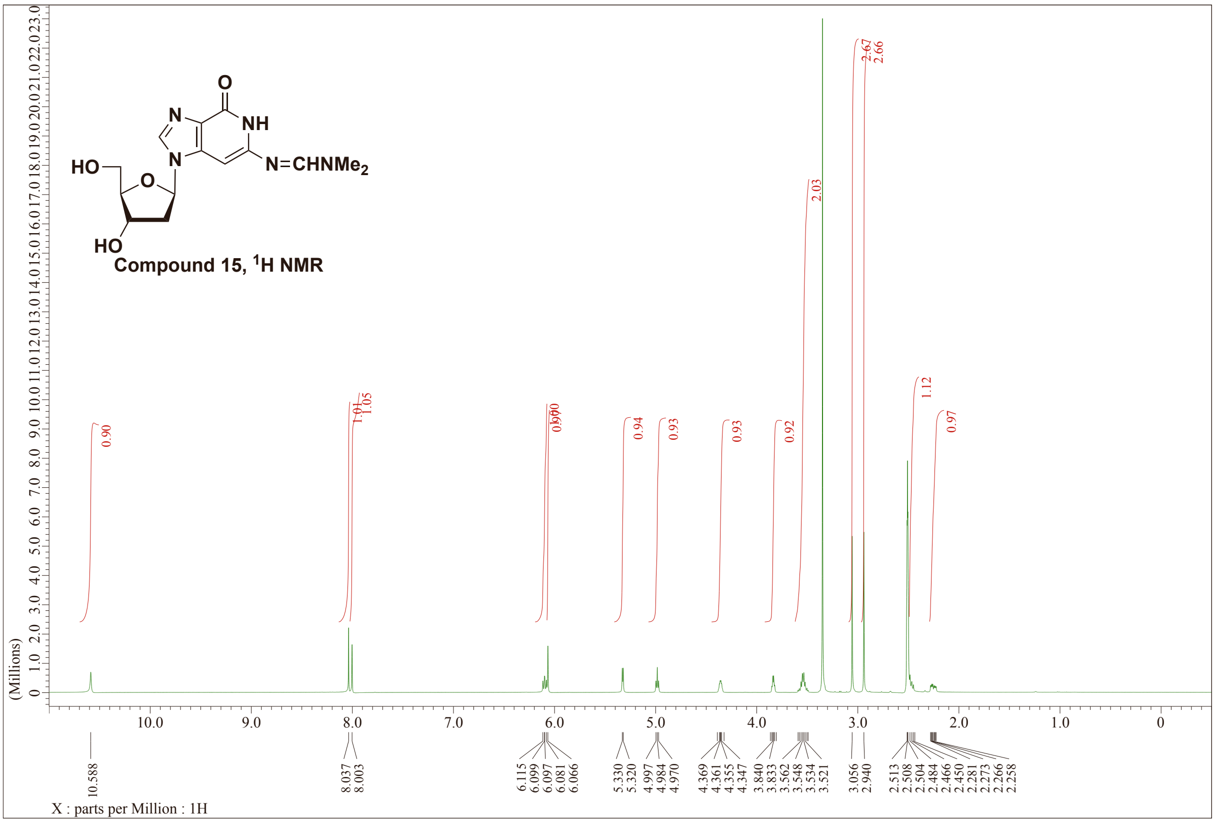

Data S12: ^{13}C NMR spectra (125 MHz, DMSO-d_6) of compound 15, related to STAR method.

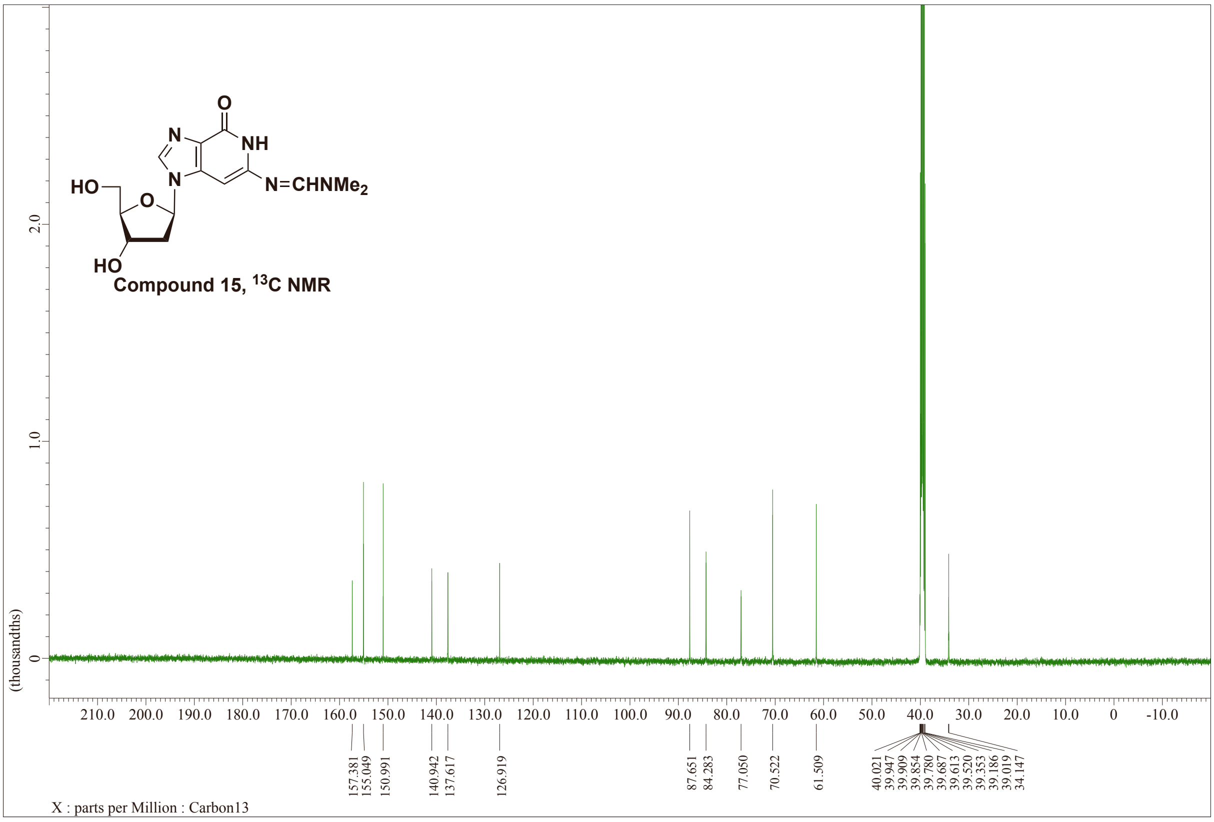

Data S13: ^{1}H NMR spectra (400 MHz, DMSO-d_6) of compound 16, related to STAR method.

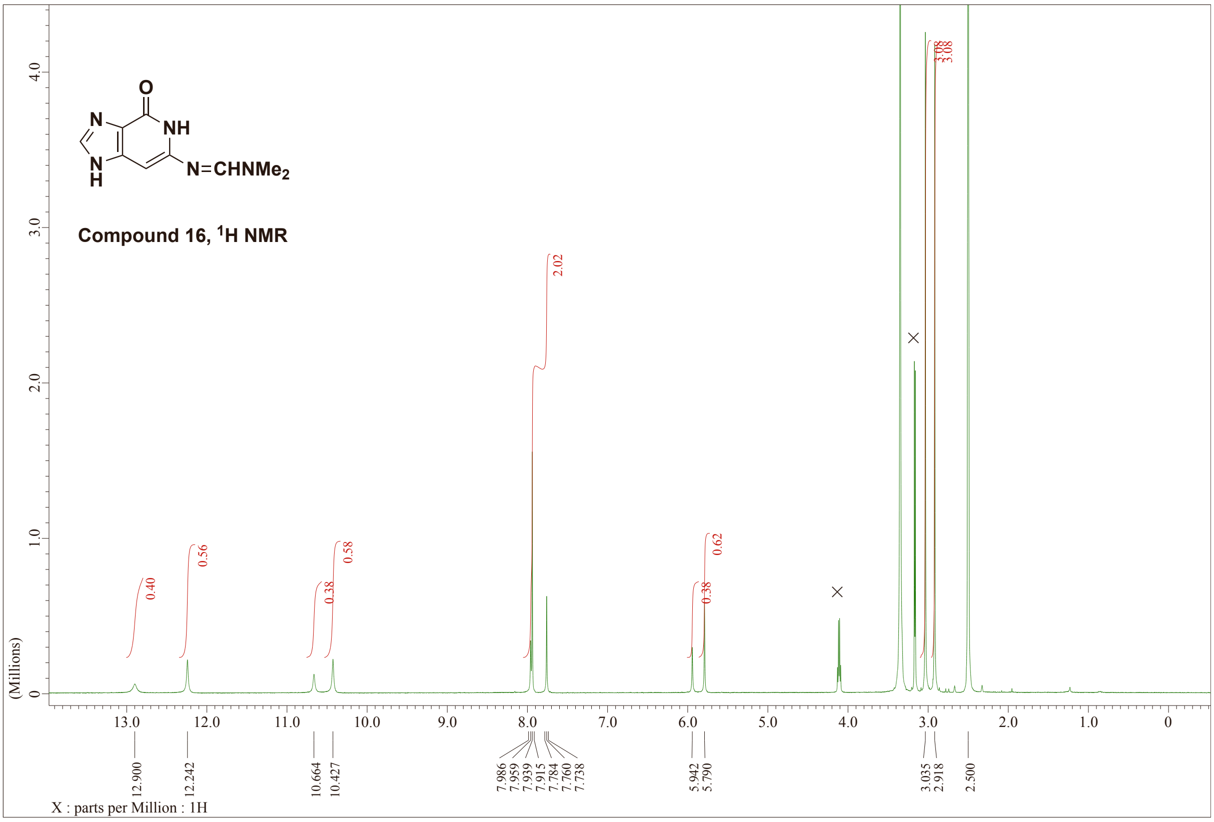

Data S14: ^{1}H NMR spectra (400 MHz, DMSO-d_6) of compound 10, related to STAR method.

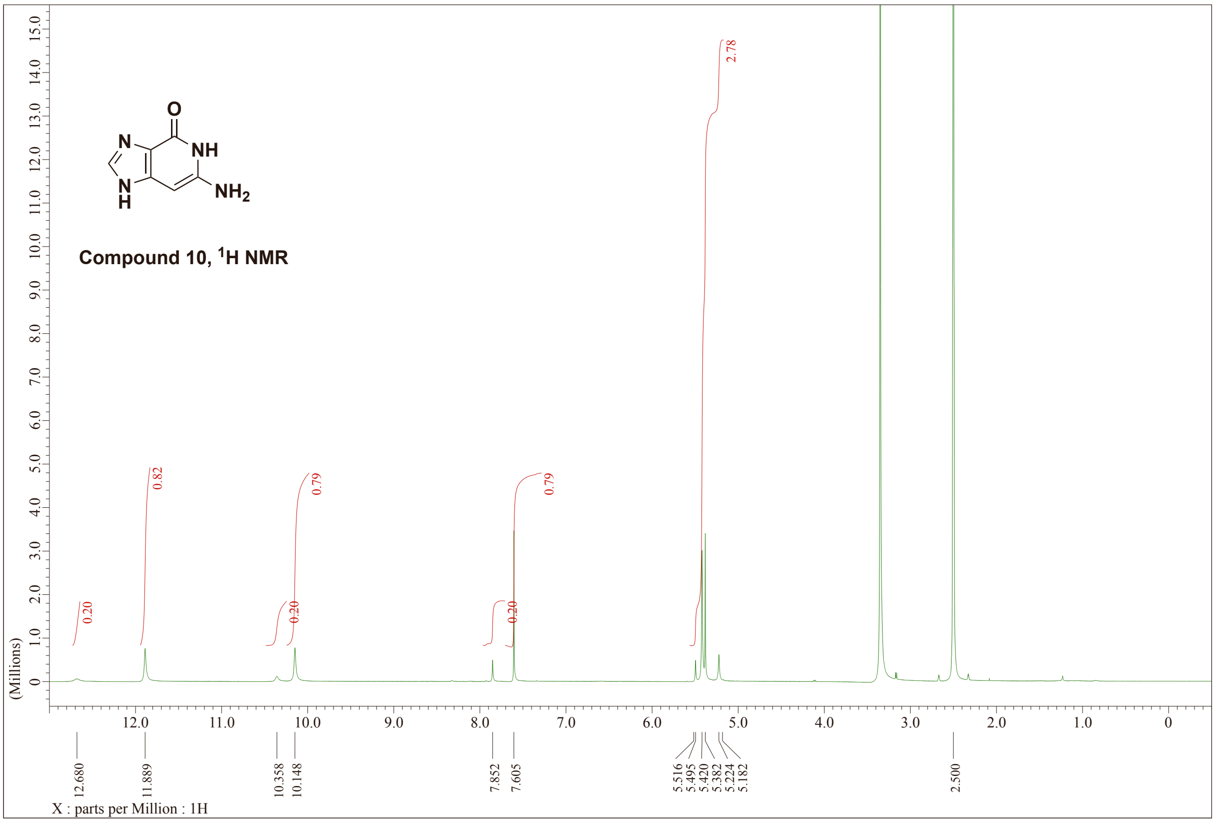

Data S15: ^{1}H NMR spectra (500 MHz, $CDCl_3$) of compound 11, related to STAR method.

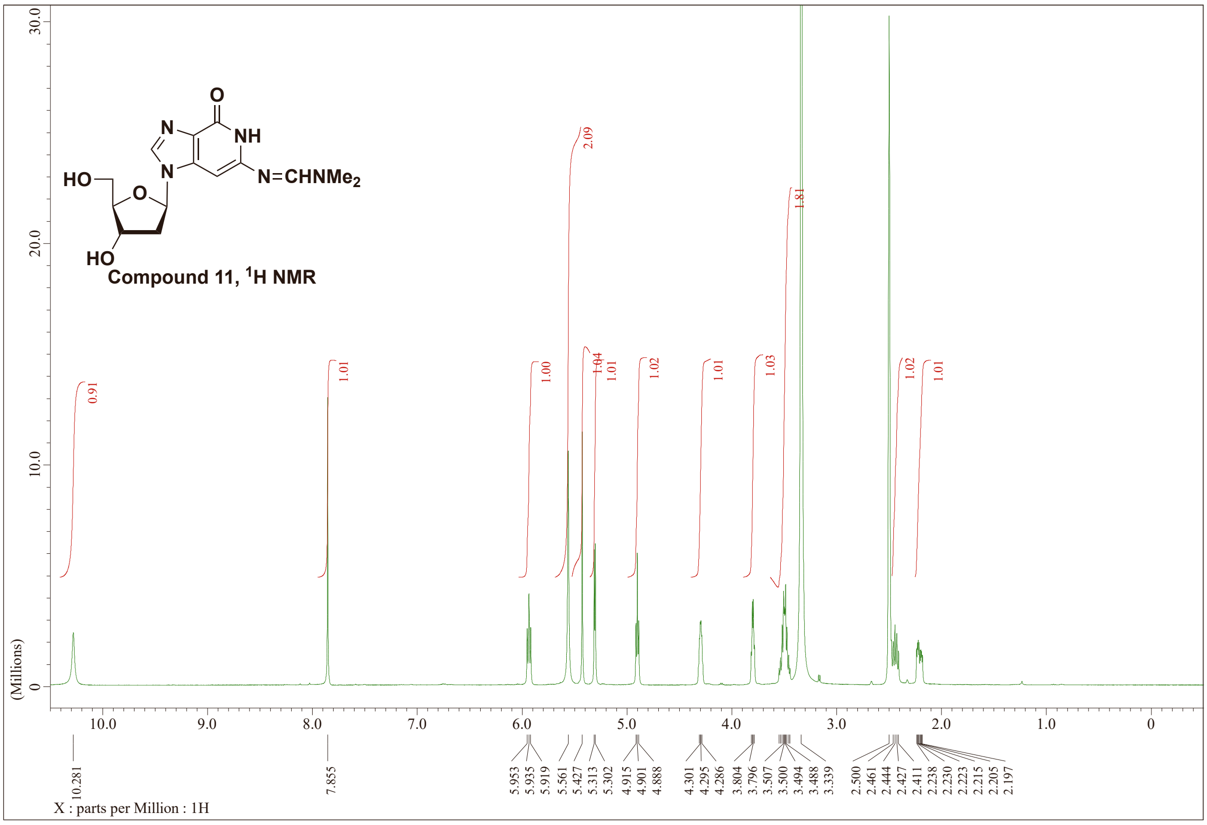

Data S16: ^{1}H NMR spectra (500 MHz, $CDCl_3$) of compound 18, related to STAR method.

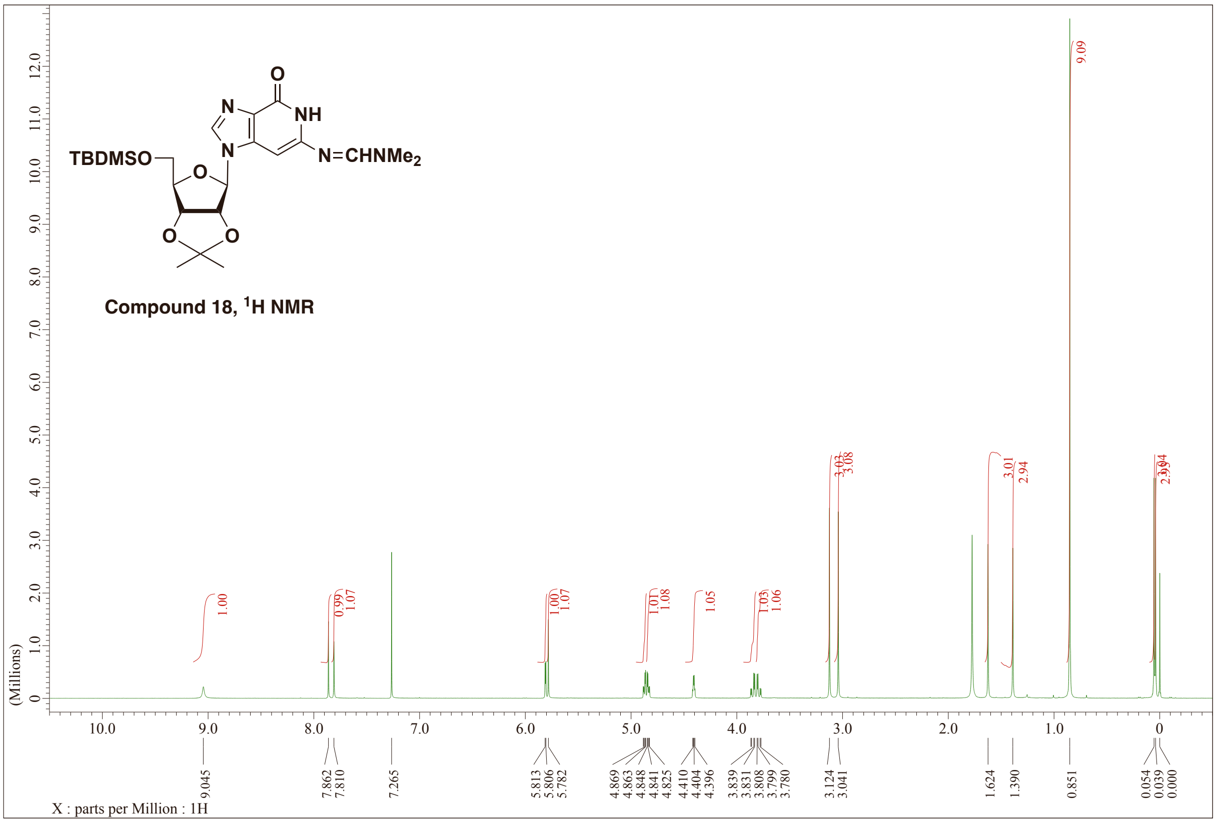

Data S17: ^{13}C NMR spectra (125 MHz, $CDCl_3$) of compound 18, related to STAR method.

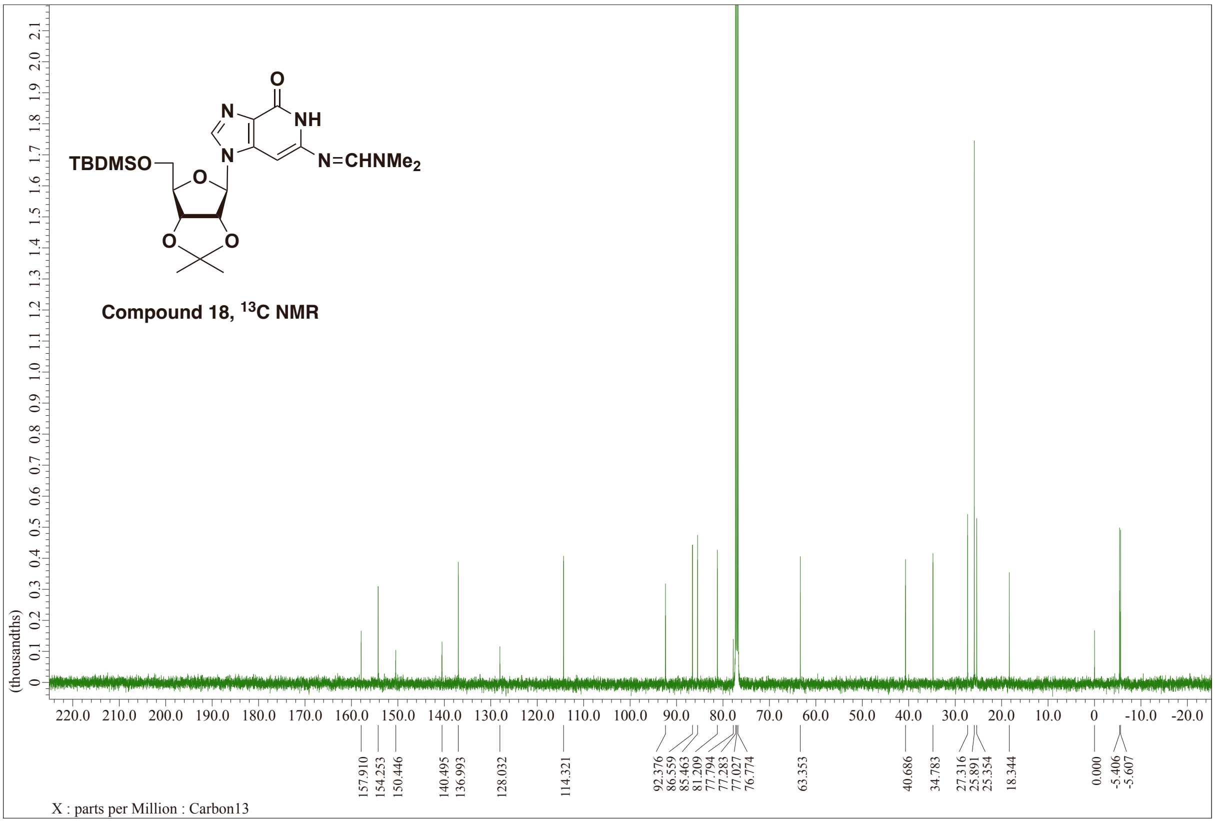

Data S18: ^{1}H NMR spectra (500 MHz, DMSO-d_6) of compound 19, related to STAR method.

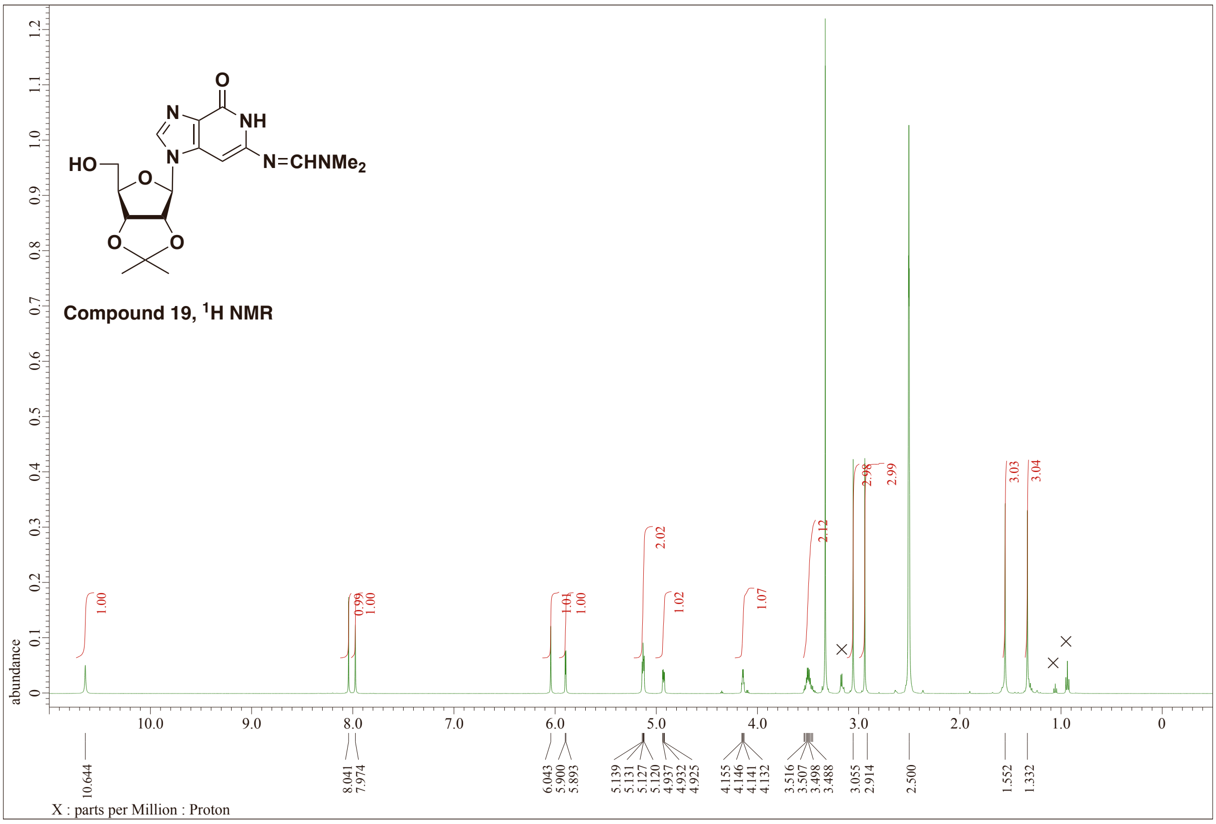

Data S19: ^{13}C NMR spectra (125 MHz, DMSO-d_6) of compound 19, related to STAR method.

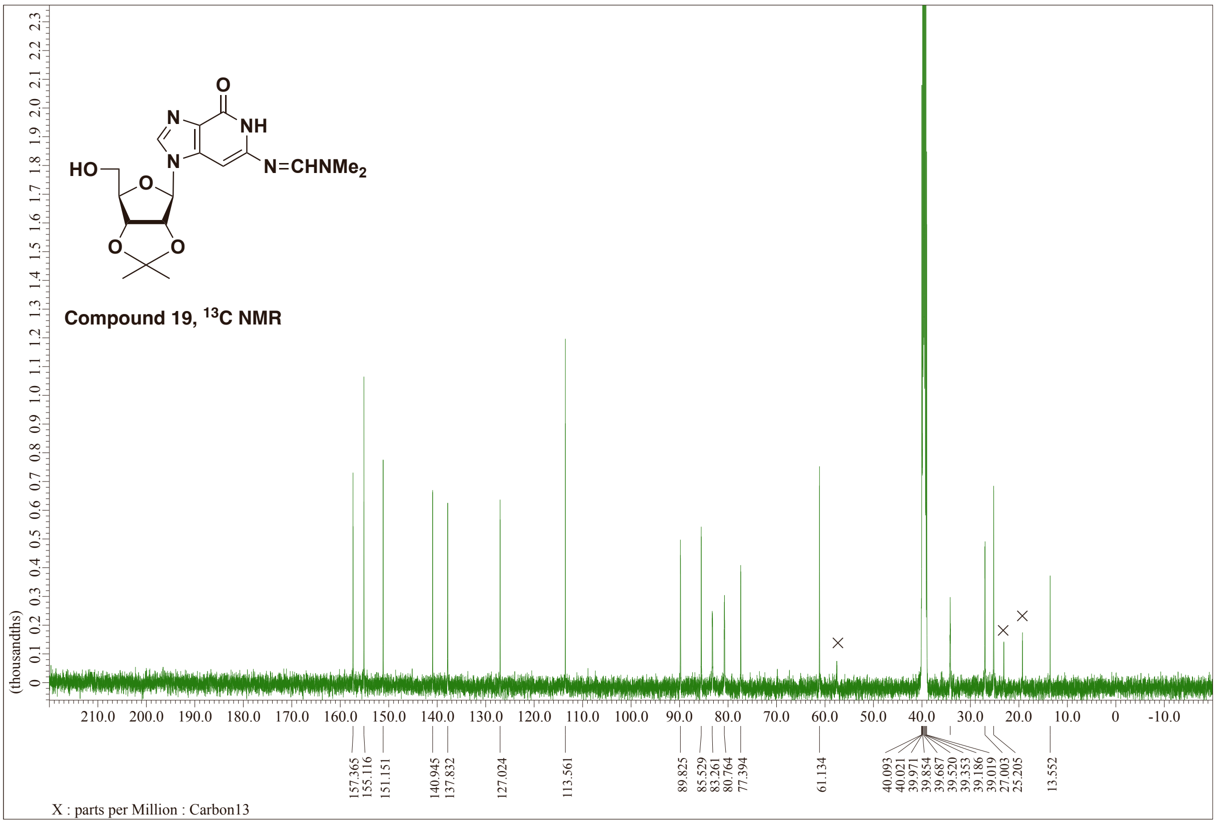

Data S20: ^{1}H NMR spectra (500 MHz, D_2O) of compound 2-TP related to STAR method.

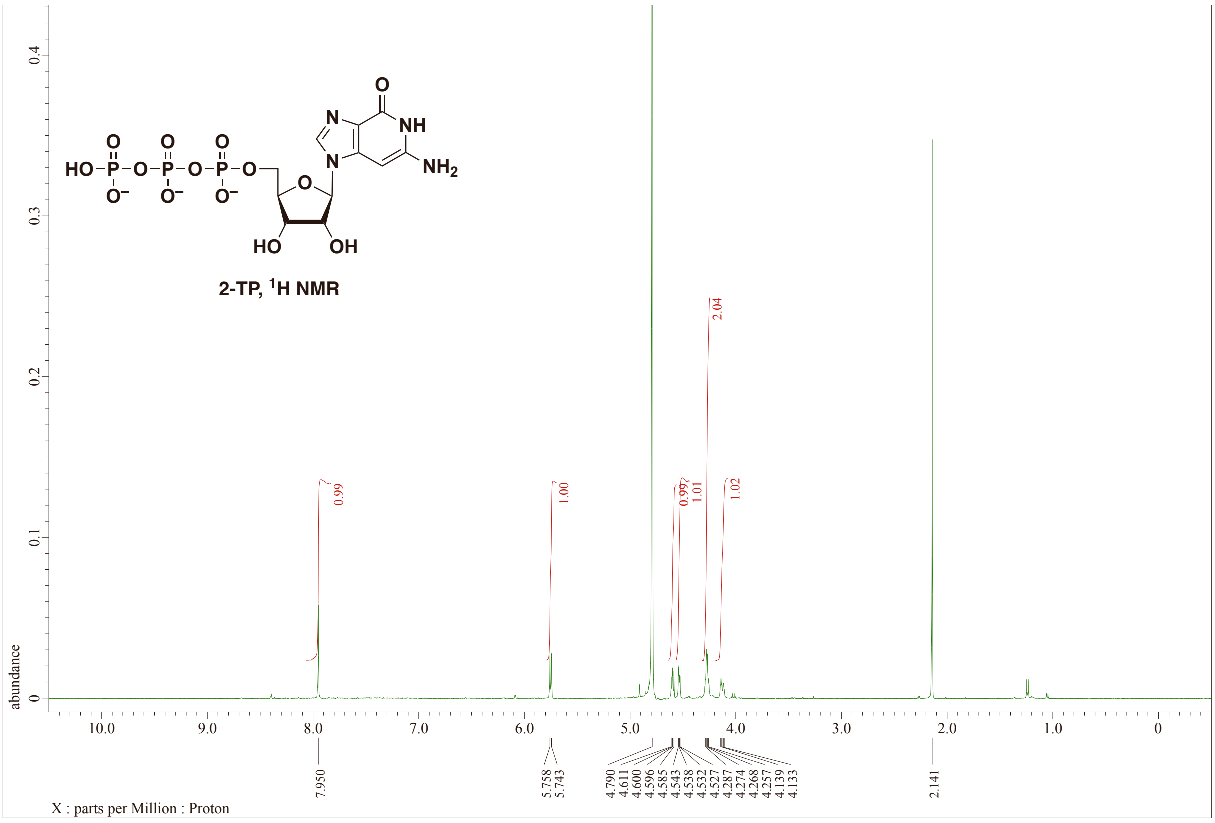

Data S21: ^{31}P NMR spectra (202 MHz, D_2O) of compound 2-TP related to STAR method.

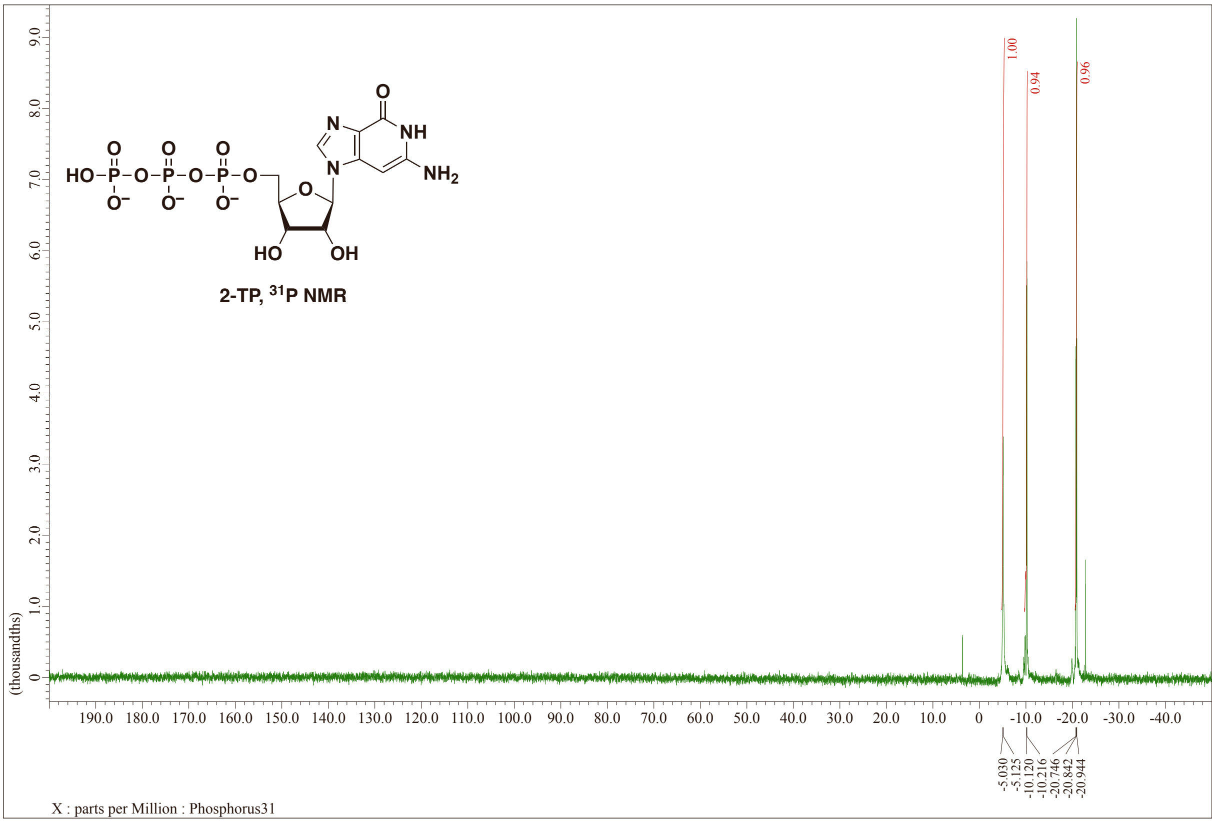

Data S22: UPLC profile of C[3]Ado (1) used in the biological assay.

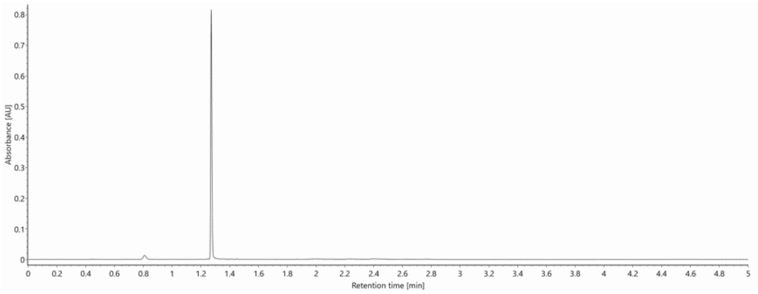

Solvent: Linear gradient of 0%–30% MeCN containing 0.1% formic acid at a flow rate of 0.4 mL/min for 5 minutes.

Data S23: UPLC profile of C[3]Guo (2) used in the biological assay.

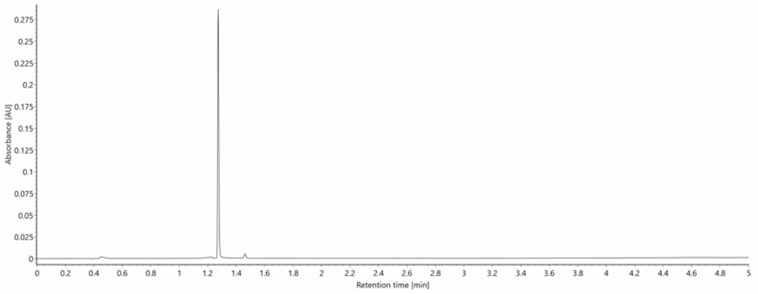

Solvent: Linear gradient of 0%–30% MeCN containing 0.1% formic acid at a flow rate of 0.4 mL/min for 5 minutes.

Data S24: UPLC profile of C[3]Ino (3) used in the biological assay.

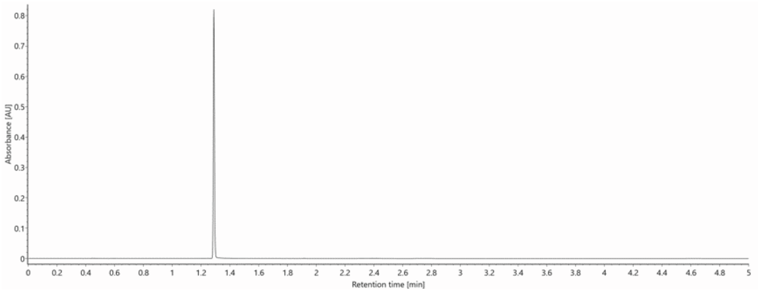

Solvent: Linear gradient of 0%–30% MeCN containing 0.1% formic acid at a flow rate of 0.4 mL/min for 5 minutes.

Data S25: UPLC profile of compound 4 used in the biological assay.

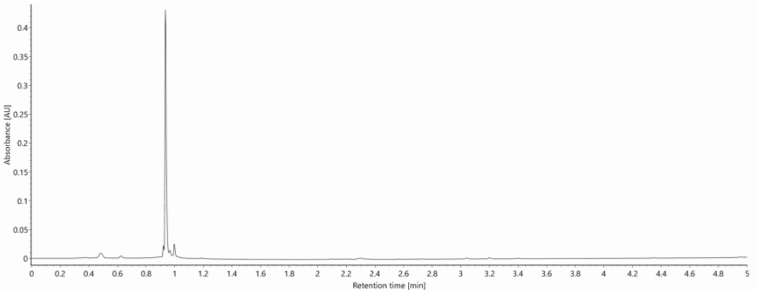

Solvent: Linear gradient of 0%–100% MeCN containing 0.1% formic acid at a flow rate of 0.4 mL/min for 5 minutes.

Data S26: UPLC profile of compound 5 used in the biological assay.

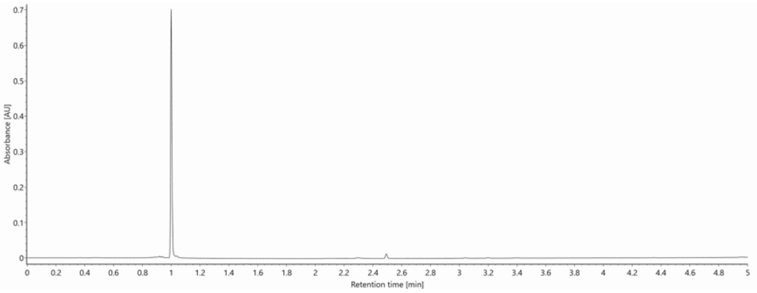

Solvent: Linear gradient of 0%–100% MeCN containing 0.1% formic acid at a flow rate of 0.4 mL/min for 5 minutes.

Data S27: UPLC profile of compound 6 used in the biological assay.

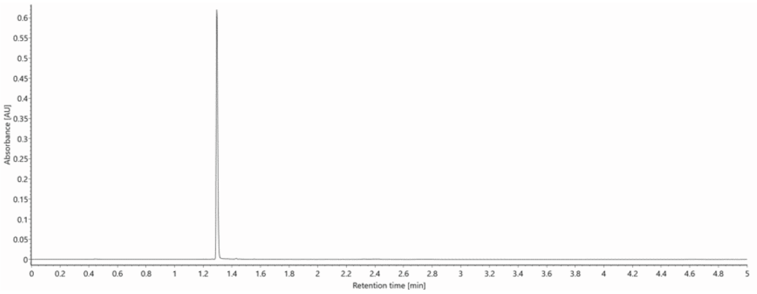

Solvent: Linear gradient of 0%–30% MeCN containing 0.1% formic acid at a flow rate of 0.4 mL/min for 5 minutes.

Data S28: UPLC profile of C^{3}Gua (10) used in the biological assay.

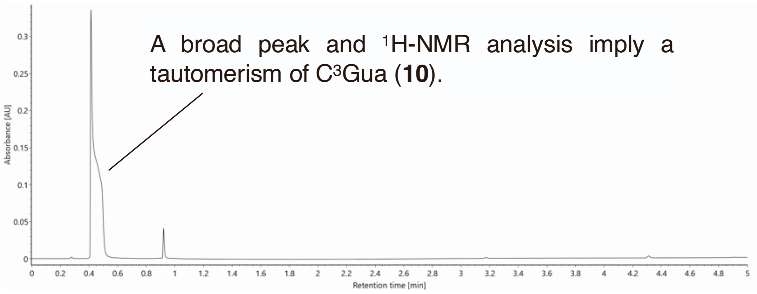

Solvent: Linear gradient of 0%–30% MeCN containing 0.1% formic acid at a flow rate of 0.4 mL/min for 5 minutes.

Data S29: UPLC profile of dC^{3}Guo (11) used in the biological assay.

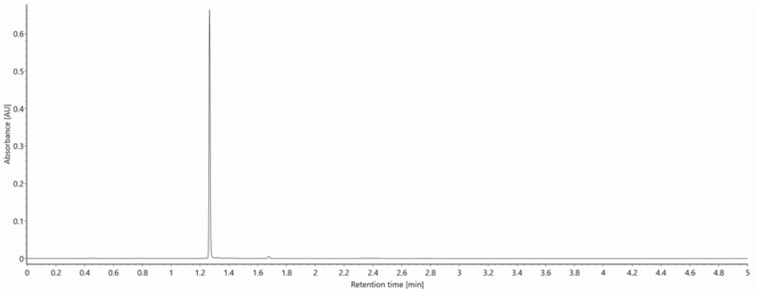

Solvent: Linear gradient of 0%–30% MeCN containing 0.1% formic acid at a flow rate of 0.4 mL/min for 5 minutes.

Data S30: UPLC profile of C^{3}Guo 5'-triphosphate (2-TP) used in the biological assay.

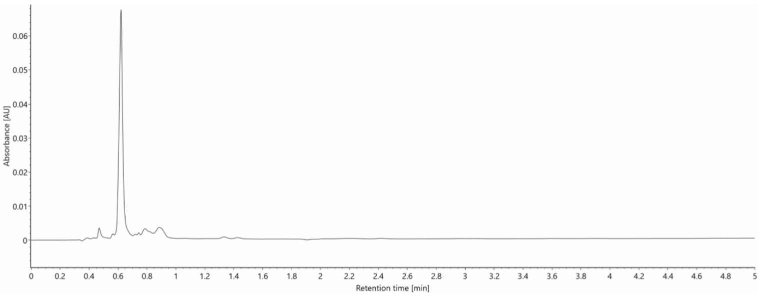

Solvent: 5% MeOH containing 10 mM triethylamine and 50 mM 1,1,1,3,3,3-Hexafluoropropan-2-ol (HFIP) at a flow rate of 0.4 mL/min for 5 minutes.
